# A Low-Complexity Hall-Based Measurement System Implementing a Dark/Illuminated Differential Estimator for Majority-Carrier Photoconductive Screening in Doped *n*-Type Silicon

**DOI:** 10.3390/ma19143016

**Published:** 2026-07-13

**Authors:** Bernardo Reyes-Durán, Carlos Álvarez-Macías, Lizbeth Salgado-Conrado, Alma Esmeralda-Gómez, Raúl Tadeo-Rosas

**Affiliations:** 1 Tecnológico Nacional de México/Instituto Tecnológico de La Laguna, Torreón 27000, Coahuila, Mexico; 2 Facultad de Ingeniería Mecánica y Eléctrica, Universidad Autónoma de Coahuila, Carretera Torreón-Matamoros, km 7.5, Torreón 27276, Coahuila, Mexico; lizbeth_salgado@uadec.edu.mx (L.S.-C.);

**Keywords:** Hall-derived estimator, majority-carrier response, comparative photoconductive screening, Van der Pauw Hall measurement, *n*-type silicon, LED illumination

## Abstract

A low-complexity Hall-based measurement system implementing a dark/illuminated differential estimator of the majority-carrier photoconductive response is assessed on *n*-type crystalline silicon wafers with contrasting doping concentrations. The setup combines a Van der Pauw configuration, permanent neodymium magnets, a characterized white LED source, and a source–measure unit to extract an apparent Hall-derived differential indicator—not a calibrated majority-carrier density—under dark and illuminated steady-state conditions. At $1.81\;\mathrm{mW}\;{\mathrm{cm}}^{-2}$, the lightly doped wafer ($N_{D}\approx 10^{14}\;{\mathrm{cm}}^{-3}$) shows a resolved response ($\Delta \sigma_{n,H}=29.61\pm 3.406\;\mathrm{mS}\;{\mathrm{cm}}^{-1}$), reported as mean ± sample standard deviation from 15 dark/light cycles. The heavily doped wafer ($N_{D}\approx 10^{17}\;{\mathrm{cm}}^{-3}$) gives a nominal below-threshold response ($\Delta \sigma_{n,H}=1.167\pm 0.140\;\mathrm{mS}\;{\mathrm{cm}}^{-1}$) from the same cycling protocol; it should be interpreted only as an unresolved trend-level indication because it lies below the instrumental detection threshold. The contrast is qualitatively consistent with the expected doping dependence, but no quantitative performance ratio is claimed for the heavily doped wafer. A Macdonald–Cuevas comparison is used only as a qualitative physical-consistency benchmark. No independent QSSPC, SSPC, μ-PCD, or carrier-resolved photo-Hall measurement was available for the same wafers; therefore, the *system* serves as a complementary low-cost screening tool for comparative trend analysis, not a replacement for calibrated photoconductance or photo-Hall techniques, and it does not constitute a new measurement principle.

## 1. Introduction

Photoconductivity, defined as the increase in the electrical conductivity of a semiconductor under illumination, is a key parameter for evaluating materials used in photovoltaic and optoelectronic applications. It provides indirect access to key transport properties such as minority-carrier lifetime (τ) and diffusion length ($L_{D}$), which govern recombination dynamics and ultimately influence device performance metrics including open-circuit voltage ($V_{\mathrm{OC}}$) and short-circuit current ($J_{\mathrm{SC}}$) [1,2]. As a result, photoconductivity establishes a direct physical link between optical absorption processes and charge-carrier transport in semiconductors.

Within the classical framework of semiconductor physics, the steady-state photoconductive response (Δσ) is expressed as the illumination-induced conductivity increase relative to the dark condition:

(1) $$\Delta \sigma =q\left(\mu_{n}\Delta n+\mu_{p}\Delta p\right)$$

where *q* is the elementary charge, $\mu_{n}$ and $\mu_{p}$ are the electron and hole mobilities, respectively, and Δn and Δp are the excess carrier densities generated by light [1,3,4]. In doped semiconductors, optical absorption produces electron–hole pairs in equal concentrations (Δn=Δp), while the equilibrium conductivity remains dominated by majority carriers due to the high carrier concentration imposed by doping [3,4]. In crystalline *n*-type silicon at room temperature, electrons exhibit a higher mobility than holes; therefore, for equal excess carrier concentrations generated under illumination, the electron-related term makes the larger contribution to the photoconductive response [5].

Under low-level injection conditions, where the excess carrier density is much smaller than the equilibrium majority-carrier concentration ($\Delta n\ll n_{0}\approx N_{D}$ for *n*-type silicon), the majority-carrier population remains nearly unchanged. In this regime, the photoconductive response can be approximated by a simplified majority-carrier expression:

(2) $$\Delta \sigma \approx q\mu_{n}\Delta n$$

which reduces analytical complexity but neglects the contribution of minority carriers and therefore provides a first-order approximation, particularly as moderate injection conditions are approached [4,6].

In the present work, the illumination-induced response is evaluated using a dark-referenced Hall-derived majority-carrier estimator, defined as

(3) $$\Delta \sigma_{n,H}=q\;\mu_{H,\mathrm{dark}}\;\Delta n_{H},$$

where $\Delta n_{H}=n_{H,\mathrm{light}}-n_{H,\mathrm{dark}}$ is the apparent Hall-derived carrier-density change between the illuminated and dark steady states. The term $\mu_{H,\mathrm{dark}}$ denotes the dark Hall mobility used as a reference mobility; it does not imply that the photoconductive response is measured in the dark. This approximation is adopted because no systematic illumination-induced mobility variation was resolved within the experimental dispersion of the present setup. Because illumination generates both electrons and holes and may modify the effective Hall coefficient, $\Delta n_{H}$ should be interpreted as an apparent Hall-derived differential metric rather than as a calibrated excess-carrier density. Therefore, $\Delta \sigma_{n,H}$ is used as a comparative Hall-derived metric and should not be interpreted as a complete carrier-resolved photoconductivity measurement. Throughout this work, $\Delta n_{H}$ is therefore treated as an empirical Hall-derived differential indicator of the illumination response rather than as a calibrated excess-carrier density, and $\Delta \sigma_{n,H}$ is used accordingly as a comparative screening metric only.

Several experimental techniques have been developed to characterize photoconductivity and carrier dynamics without requiring complete device fabrication. Among the most widely adopted approaches, steady-state photoconductance (SSPC) and quasi-steady-state photoconductance (QSSPC) methods enable extraction of effective minority-carrier lifetime over broad injection ranges under controlled illumination conditions [7,8,9,10]. These methods have been extensively applied to crystalline silicon and emerging photovoltaic materials, providing insight into recombination mechanisms, injection-dependent lifetime behavior, and surface versus bulk recombination contributions. In parallel, advanced techniques such as the photo-Hall effect provide the additional advantage of decoupling carrier density and mobility under illumination, enabling a more complete carrier-transport characterization [11,12,13,14,15]. Recent developments have extended these capabilities to electronic trap detection [12], simultaneous multi-mode lifetime and mobility extraction [13,16], broadband time-resolved photoconductance [17], and light-induced colossal magnetoresistance effects in silicon [14], collectively raising the state of the art for carrier-transport characterization in photovoltaic materials.

Despite their accuracy, many of these established techniques require complex experimental configurations. QSSPC measurements typically rely on calibrated flash or modulated illumination sources with well-defined temporal profiles, combined with synchronized data acquisition and specialized instrumentation. Similarly, modern photo-Hall implementations often involve non-trivial magnetic-field control, lock-in detection, and multi-parameter fitting procedures, increasing both cost and experimental complexity [9,10,11]. These requirements can limit accessibility in low-resource laboratory environments, particularly when only comparative material screening is required.

In this context, Hall-based measurements offer a practical pathway for comparative estimation of illumination-induced majority-carrier changes, as they inherently provide information on carrier concentration and mobility. Motivated by this capability, the present work implements and assesses a low-complexity steady-state Hall-based method using permanent magnets, continuous LED illumination, and a standard source–measure unit. The proposed approach is intended as an accessible framework for comparative photoconductive analysis of doped silicon wafers. The scope of the present screening system relative to established photoconductance and photo-Hall techniques is summarized in Table 1. Within its intended operating regime, the method preserves sensitivity to doping-dependent photoconductive trends under steady-state illumination.

The contribution of this work lies in the implementation and scope-defining assessment of a dark/illuminated two-state Hall-differential estimator for low-complexity, majority-carrier-based comparative photoconductive screening. The two states correspond to a dark baseline measurement and a steady-state measurement under continuous white-LED illumination, from which the apparent Hall-derived carrier-density change is obtained as $\Delta n_{H}=n_{H,\mathrm{light}}-n_{H,\mathrm{dark}}$. This differential quantity is then converted into the dark-referenced Hall-derived estimator $\Delta \sigma_{n,H}=q\mu_{H,\mathrm{dark}}\Delta n_{H}$, providing a practical comparative metric rather than a complete carrier-resolved photoconductivity measurement. The contribution is a specific low-cost system implementation and a defined operating scope; it does not propose a new measurement principle.

In particular, the study combines Hall-factor sensitivity analysis, detection-limit evaluation, Van der Pauw consistency checking, and explicit identification of the detection-threshold regime, thereby defining the practical scope of the method for low-resource laboratory environments.

Because independent QSSPC, SSPC, µ-PCD, or carrier-resolved photo-Hall measurements were not available for the same wafers, the present analysis focuses on qualitative consistency in doping dependence and injection behavior. The experimental trends are therefore discussed in the context of reported photoconductance and photo-Hall studies on crystalline silicon with comparable doping ranges. As a consequence, three quantities cannot be established from the present dataset: (i) the absolute accuracy of the estimator relative to a calibrated reference, (ii) whether its response scales correctly with excess carrier density, and (iii) whether the resolved trend originates from photoconductivity or from a Hall-coefficient artifact. The contribution is therefore a low-cost system implementation and scope-defining assessment, not a new measurement principle or a validated substitute for calibrated photoconductance or photo-Hall methods.

## 2. Materials and Methods

### 2.1. Experimental Setup

The experimental system was based on the Van der Pauw technique combined with classical Hall measurements under controlled steady-state illumination. The configuration is methodologically related to photoconductance and photo-Hall approaches, but it does not provide carrier-resolved transport information [11,18]. Electrical characterization of the doped silicon wafers was performed using a four-point ECOPIA SPCB-01 sample holder (ECOPIA, Anyang, Republic of Korea) connected to a Keithley 2450 source–measure unit (SMU; Keithley Instruments, Cleveland, OH, USA), following standard procedures for resistivity and Hall measurements in single-crystal semiconductors [19,20].

The investigated *n*-type silicon wafers had nominal thicknesses of $t_{1}=525\;\mu m$ (0.0525cm) and $t_{2}=500\;\mu m$ (0.0500cm), which were used in the calculation of resistivity, Hall coefficient, carrier density, and mobility [1,4]. To minimize electrical noise, thermoelectric offsets, and parasitic voltage contributions—particularly critical in low-signal Hall measurements—the complete setup was enclosed inside a Faraday cage with dimensions of 40.5×35×29cm [20].

**Figure 1:**
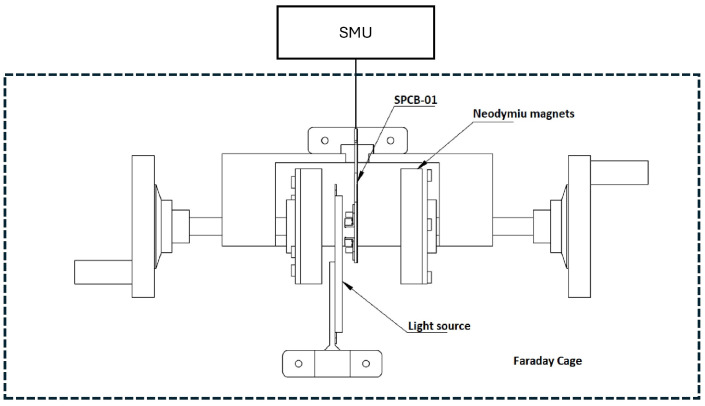
Experimental setup showing SPCB-01 sample holder between N52 permanent magnets, white LED source, Faraday cage, and Keithley 2450 SMU.

As illustrated in Figure 1, each wafer was mounted in the SPCB-01 holder and positioned between two N52-grade neodymium permanent magnets (Innovatec, Monterrey, Mexico), producing a static transverse magnetic field. The magnetic field was adjustable between 143 and 210mT through a worm-screw positioning mechanism and measured directly at the wafer plane using a calibrated gaussmeter (WT10A; Shenzhen Graigar Technology Co., Ltd., Shenzhen, China) [20,21]. For irradiance-dependent measurements, a nominal value of B=200.0±0.4mT was selected to ensure sufficient Hall-voltage resolution while maintaining experimental simplicity. The expected linear dependence of Hall voltage on magnetic field in the classical regime supports the use of a fixed magnetic field within this range [4,20].

Hall voltage measurements were performed using magnetic-field polarity reversal to suppress offset voltages associated with contact misalignment, thermoelectric effects, and illumination-induced parasitic contributions [20,21]. The transverse voltage was recorded under both magnetic polarities, and the Hall component was extracted as the antisymmetric part of the measured signal. Additional current-dependent measurements under both dark and illuminated conditions supported the linearity and sign consistency of the Hall response. Nevertheless, residual uncertainties could still arise from parasitic photovoltages, lateral photoconductive gradients, and thermophotovoltaic effects under illumination [11,18].

**Figure 2:**
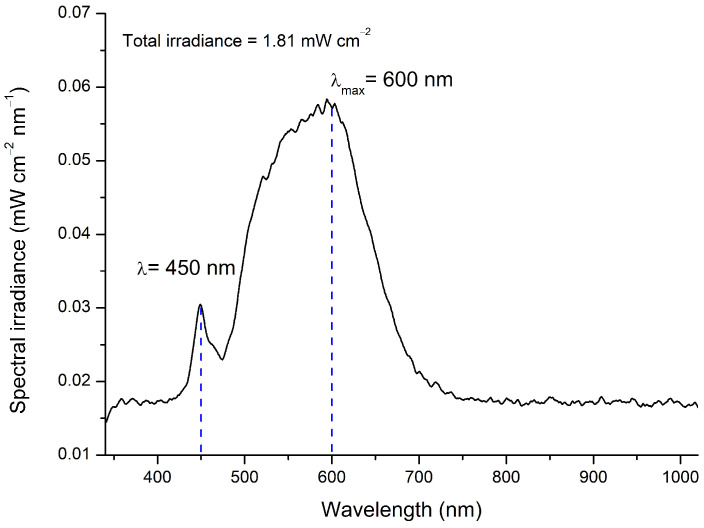
Spectral irradiance of the white LED source measured at the wafer plane using an Ocean Optics USB2000+ spectrometer with cosine corrector (total irradiance: $1.81\;\mathrm{mW}\;{\mathrm{cm}}^{-2}$).

Illumination was provided by a continuous white LED source with a 4mm emitting package positioned above the wafer. The irradiance at the sample plane was measured as $1.81\;\mathrm{mW}\;{\mathrm{cm}}^{-2}$. The emission spectrum was experimentally characterized using an Ocean Optics USB2000+ spectrometer (Ocean Optics, Dunedin, FL, USA) equipped with an SM905 cosine corrector, as shown in Figure 2. The temporal stability of the LED was quantified by a short-term instability of STI=1.87%, measured over the duration of a single measurement cycle (3–5s), while the spatial non-uniformity over the measurement area was measured as 7.46%. These parameters define the illumination conditions used for controlled comparative measurements. No formal ASTM solar-simulator classification is claimed, since this would require standardized evaluation of spectral match, spatial uniformity, and temporal stability under reference conditions [22].

Due to the wavelength-dependent absorption coefficient of crystalline silicon, optical generation under broadband white LED illumination is inherently non-uniform across the wafer thickness. Shorter wavelengths are absorbed near the surface, whereas longer wavelengths penetrate deeper into the bulk [23,24]. Consequently, the extracted Hall-response variation represents an effective volume-averaged electrical response, influenced by depth-dependent generation and surface recombination effects [1,6].

Overall, the use of permanent magnets and continuous LED illumination enables a reduced-complexity implementation without the need for electromagnets, AC excitation, or lock-in detection, in contrast to more advanced photo-Hall and light-induced magnetotransport methods [11,18]. Although the simplified configuration does not provide full carrier-resolved characterization, it enables practical comparative analysis between wafers with different doping levels using standard Hall and Van der Pauw measurement principles [4,20].

### 2.2. Measurement Procedure

All measurements were performed at a controlled temperature of 300±0.5K on two *n*-type crystalline silicon wafers exhibiting strongly different dark resistivities. Wafer 1 had a thickness of 525±1μm and a resistivity of 16.15Ω·cm, whereas wafer 2 had a thickness of 500±1μm and a resistivity of 0.081Ω·cm. These values correspond to significantly different doping concentrations, enabling comparative analysis under identical experimental conditions. The expected resistivity–dopant concentration relationship was used as a consistency reference for the extracted Hall-derived carrier concentrations [25]. The donor concentration $N_{D}$ was not measured directly; it was extracted from the measured Van der Pauw resistivity through this relation and cross-checked against the Hall-derived concentration. Combining the resistivity dispersion (Table 2) with the stated accuracy of the ρ–$N_{D}$ relation gives $u\left(N_{D}\right)/N_{D}\approx 5\%$, which propagates to an injection-ratio uncertainty of $\Delta n_{H}/N_{D}=0.48\pm 0.03$ for wafer 1.

Prior to the electrical measurements, both wafers were chemically cleaned using a standard RCA (Radio Corporation of America) procedure in order to remove organic residues and surface contaminants that could degrade contact quality or influence recombination behavior [26]. After cleaning, the wafers were mounted in the ECOPIA SPCB-01 holder and electrically contacted in a four-point Van der Pauw configuration.

**Figure 3:**
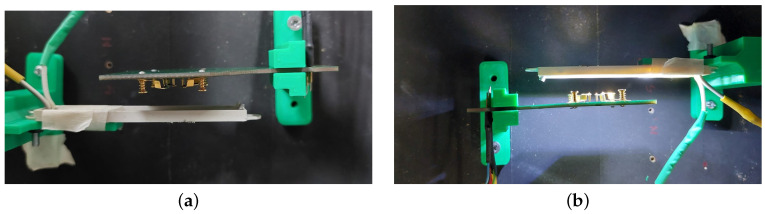
Van der Pauw resistivity configuration under (**a**) dark and (**b**) illuminated conditions ($1.81\;\mathrm{mW}\;{\mathrm{cm}}^{-2}$).

Resistivity measurements were performed using the Van der Pauw method with a Keithley 2450 source–measure unit (SMU). A DC excitation current of I=50μA was applied while the corresponding voltages were recorded in the standard four-terminal configuration. Measurements were conducted under both dark and illuminated conditions, as illustrated in Figure 3. This current level was selected to ensure adequate voltage resolution while minimizing Joule heating effects [19,21].

**Figure 4:**
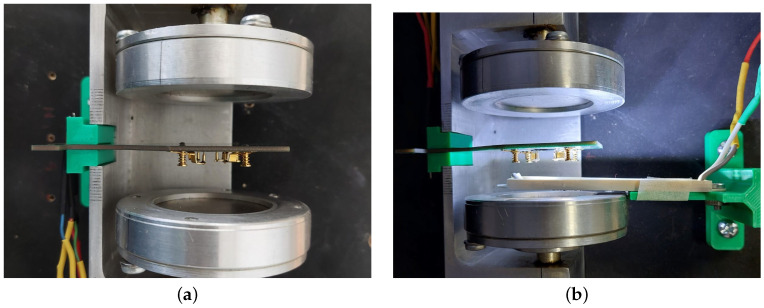
Hall voltage measurement configuration under (**a**) dark and (**b**) illuminated conditions ($1.81\;\mathrm{mW}\;{\mathrm{cm}}^{-2}$) using N52 permanent magnets.

Hall-effect measurements were subsequently performed using the same contact configuration in order to determine the Hall coefficient, majority-carrier density, and Hall mobility. The Hall voltage was measured by applying the transverse magnetic field and recording the corresponding transverse voltage. To mitigate voltage offsets caused by contact misalignment, thermoelectric effects, and illumination-induced parasitic voltages, the magnetic-field polarity was reversed and the Hall voltage was extracted as the antisymmetric component of the measured signal, as illustrated in Figure 4 [20,21]. The linearity of the Hall response was assessed through additional current-dependent sweeps under both dark and illuminated conditions.

The Hall coefficient was calculated using the standard relation:

(4) $$R_{H}=\frac{V_{H}t}{IB}$$

where $V_{H}$ is the Hall voltage, *t* is the wafer thickness, *I* is the applied current, and *B* is the magnetic field. The carrier density was then obtained as a nominal Hall-derived majority-carrier concentration, $n_{H}^{\left(1\right)}$, by assuming $r_{H}=1$ as the reference case. Since the Hall factor in doped silicon can deviate from unity depending on dopant concentration and scattering mechanism, the reported carrier densities represent effective electrical carrier concentrations [4,5]. A Hall-factor sensitivity analysis was therefore included to evaluate the effect of plausible $r_{H}$ variations on $n_{H}$, $\Delta n_{H}$, and $\Delta \sigma_{n,H}$. The sign of $R_{H}$ confirmed *n*-type conduction for both wafers. The Hall mobility was calculated by combining the Hall coefficient with the measured resistivity according to standard formulations [4,5].

A two-state measurement protocol was implemented consisting of a baseline characterization under dark conditions followed by measurements under continuous illumination using the white LED source at an irradiance of $1.81\;\mathrm{mW}\;{\mathrm{cm}}^{-2}$. For the Hall-derived photoconductive estimates reported in the main results, the excitation current was fixed at 50μA, the magnetic field at approximately 200mT, and the acquisition time was maintained between 3 and 5s in order to reduce thermal drift and minimize heating artifacts [20,21]. The light-induced apparent Hall-derived carrier-density change was estimated from the difference between the illuminated and dark Hall-derived carrier concentrations as follows:

(5) $$\Delta n_{H}=n_{H,\mathrm{light}}-n_{H,\mathrm{dark}}$$

where $n_{H,\mathrm{light}}$ and $n_{H,\mathrm{dark}}$ are the illuminated and dark Hall-derived carrier concentrations, respectively. This difference is treated as an effective Hall-response variation used for comparative photoconductive analysis.

Measurement repeatability and short-term stability were evaluated using two complementary datasets. First, magnetic-field sweeps over six field values and current-dependent sweeps over ten current values were performed under dark and illuminated conditions. Second, the dark/light two-state protocol was repeated after three independent sample mountings, with 5 dark/light cycles per mounting, giving 15 cycles for each wafer. In the Hall measurements, magnetic-field polarity reversal was applied for each condition, and the Hall voltage was obtained from the antisymmetric voltage component. The resulting data were used to calculate the mean, sample standard deviation, and coefficient of variation for the measured and derived quantities reported in Table 2 and Table 3. The remounting dataset quantifies short-term mounting and cycling reproducibility for the present setup, while longer-term day-to-day drift and contact-aging effects remain outside the scope of this study.

For the estimation of photoconductive response, the electron mobility under illumination was assumed equal to the dark Hall mobility, $\mu_{H,\mathrm{dark}}$, since no systematic mobility variation was resolved between dark and illuminated conditions within the experimental dispersion of the present setup. This assumption provides a first-order approximation that remains reasonable under low-to-moderate injection conditions [5,27].

Based on this approach, the photoconductive response is reported as a Hall-based majority-carrier estimator that does not explicitly include minority-carrier contributions. This distinction is relevant when comparing the present results with full photoconductance models or carrier-resolved techniques [8,28].

Potential sources of error in Hall measurements under illumination include parasitic photovoltages, lateral carrier gradients, and contact-related photo-thermoelectric effects. These contributions were mitigated through the use of symmetric Van der Pauw geometry, electromagnetic shielding, magnetic-field reversal, and assessment of current linearity. Residual contributions were treated as sources of systematic uncertainty inherent to the simplified steady-state configuration.

Finally, uncertainties associated with wafer thickness, current, magnetic field, Hall voltage, and resistivity were propagated to estimate the uncertainty in derived parameters, including $R_{H}$, $n_{H}$, $\mu_{H}$, $\Delta n_{H}$, and $\Delta \sigma_{n,H}$ [29]. The magnetic-field and current-sweep data were used to estimate short-term statistical dispersion, while instrument specifications were considered for systematic type-B contributions. The experimentally obtained photoconductive response was compared with the Macdonald–Cuevas model [30,31], which incorporates carrier trapping and recombination mechanisms in crystalline silicon. The steady-state photoconductance Δσ was computed using the majority- and minority-carrier photoconductance expression of Macdonald et al. [30] (their Equation (1) with the bias-light correction term set to zero, corresponding to steady-state illumination without a background bias light), evaluated over the irradiance range from 0 to $1.81\;\mathrm{mW}\;{\mathrm{cm}}^{-2}$ using the carrier lifetime parametrisation of Macdonald and Cuevas [31]. This comparison served as a qualitative physical-consistency benchmark rather than a direct calibrated reference comparison.

### 2.3. Uncertainty Analysis

The uncertainty analysis was performed by propagating the primary measured quantities involved in the Hall-based photoconductivity estimation, namely the Hall voltage $V_{H}$, excitation current *I*, magnetic field *B*, wafer thickness *t*, and Van der Pauw resistivity ρ. A first-order root-sum-square (RSS) method was applied to estimate the combined standard uncertainty of the derived parameters [29].

For a set of *N* repeated measurements, the mean value, sample standard deviation, and coefficient of variation were calculated as

(6) $$\bar{x}=\frac{1}{N}\sum_{i=1}^{N}x_{i}$$

(7) $$s_{x}=\sqrt{\frac{1}{N-1}\sum_{i=1}^{N}{\left(x_{i}-\bar{x}\right)}^{2}}$$

(8) $$CV=100\frac{s_{x}}{|\bar{x}|}$$

In the results reported below, *N* takes the value of 6 for magnetic-field sweeps, 10 for current sweeps, and 5 or 15 for dark/light cycling measurements, as detailed in Table 2 and Table 3.

The relative uncertainty of the Hall coefficient $R_{H}$ was obtained by combining the independent uncertainty contributions associated with $V_{H}$, *t*, *I*, and *B*, according to standard uncertainty propagation rules [29]. Since the Hall-derived carrier density $n_{H}$ is inversely proportional to $R_{H}$, its relative uncertainty is equivalent to that of the Hall coefficient. In the case of the Hall mobility, additional uncertainty was included through the propagated contribution of resistivity ρ, since mobility is derived from both $R_{H}$ and ρ [4,5].

The uncertainty in the apparent Hall-derived carrier-density change $\Delta n_{H}$ was estimated from the independent uncertainties of the dark and illuminated Hall-derived carrier densities. Subsequently, this uncertainty was propagated to the Hall-based majority-carrier photoconductive estimator $\Delta \sigma_{n,H}$, which depends on $\Delta n_{H}$ and the assumed constant mobility $\mu_{H,\mathrm{dark}}$ [29].

Using representative experimental values of u(B)/B≈0.2%, u(I)/I≈0.05%, and a thickness uncertainty of u(t)=1μm, together with the observed repeatability of Hall-voltage measurements, the relative combined standard uncertainty of the Hall coefficient due only to instrumental RSS contributions was estimated to lie within the range of 0.28–0.30%. This corresponds to an expanded instrumental uncertainty of approximately 0.6% (coverage factor k=2) for the Hall-derived carrier density $n_{H}$. Because this estimate excludes systematic contributions associated with the Hall factor, illumination non-uniformity, thermal drift, sample remounting, contact alignment, magnetic-field spatial variation, and residual parasitic photovoltages [29], the expanded uncertainty reported here should not be interpreted as the total uncertainty of the method, but as a lower-bound instrumental contribution. Accordingly, the reported 0.28–0.30% RSS contribution and the corresponding expanded uncertainty of approximately 0.6% represent only the instrumental uncertainty component.

Consequently, the uncertainty analysis is separated into an instrumental RSS component and a set of systematic uncertainty contributions. The instrumental component describes the repeatability and propagation of directly measured electrical quantities, whereas the systematic component accounts for effects that may bias the Hall-derived estimator but were not independently calibrated in the present setup. These systematic terms are particularly relevant for the heavily doped wafer, where the light-induced differential signal lies below the detection threshold.

The detection-threshold criterion (${\mathrm{SNR}}_{\Delta n}=3$) used to classify the wafer 2 response is a propagated Type B instrumental uncertainty criterion based on $u_{c}\left(n_{H}\right)/n_{H}\approx 0.30\%$; it does not represent a noise-floor or digitizer-resolution limit (see Section 2.4).

Finally, the illumination-related parameters reported in Table 4 are provided as descriptors of the experimental conditions and as systematic contributions affecting the irradiance-dependent response. They are not intended to represent an ASTM solar simulator classification, since standardized spectral match, spatial uniformity, and temporal stability requirements were not evaluated under reference testing conditions [22].

### 2.4. Detection Limit and Signal-to-Noise Considerations

The detectability of the illumination-induced Hall-derived carrier-density variation was evaluated by comparing the extracted excess carrier concentration $\Delta n_{H}$ with the propagated uncertainty of the dark and illuminated carrier density measurements. Since the proposed method relies on a differential measurement between two steady states (dark and illuminated), the minimum detectable signal is fundamentally limited by the combined dispersion and systematic uncertainty of the Hall-derived carrier density $n_{H}$ [29].

To formalize this assessment, a signal-to-noise ratio for the apparent Hall-derived carrier-density change was defined as

(9) $${\mathrm{SNR}}_{\Delta n}=\frac{|\Delta n_{H}|}{u_{c}\left(\Delta n_{H}\right)},$$

where $u_{c}\left(\Delta n_{H}\right)$ is the combined standard uncertainty of the apparent Hall-derived carrier-density change:

(10) $$u_{c}\left(\Delta n_{H}\right)=\sqrt{u_{c}^{2}\left(n_{H,\mathrm{light}}\right)+u_{c}^{2}\left(n_{H,\mathrm{dark}}\right)}.$$

The response was classified as resolved when ${\mathrm{SNR}}_{\Delta n}>3$, indicative when $1<{\mathrm{SNR}}_{\Delta n}\leq 3$, and unresolved or interpretable only as an upper-limit response when ${\mathrm{SNR}}_{\Delta n}\leq 1$. The detection threshold was therefore defined as ${\mathrm{SNR}}_{\Delta n}=3$: a response was resolved only when $|\Delta n_{H}|\;>3\;u_{c}\left(\Delta n_{H}\right)$, where $u_{c}\left(\Delta n_{H}\right)$ is the combined standard (1σ) uncertainty propagated from the instrumental RSS budget of Table 4 ($u_{c}\left(n_{H}\right)/n_{H}\approx 0.30\%$). The threshold was thus a propagated-uncertainty (Type B instrumental) criterion, not a digitizer resolution or a noise-floor measurement. For wafer 2 it corresponded to a minimum detectable $|\Delta n_{H}|\;\approx 1.2\;\times \;10^{15}\;{\mathrm{cm}}^{-3}$, well above the observed $9.13\times 10^{12}\;{\mathrm{cm}}^{-3}$. The larger remounting and cycling dispersion of Table 3 characterizes practical reproducibility, whereas the instrumental floor defined here characterizes detectability.

Using the quantified instrumental RSS component alone (excluding illumination non-uniformity), with $u_{c}\left(n_{H}\right)/n_{H}\approx 0.30\%$, the estimated values were ${\mathrm{SNR}}_{\Delta n}\approx 88$ for wafer 1 and ${\mathrm{SNR}}_{\Delta n}\approx 2.3\times 10^{-2}$ for wafer 2.

The contribution of the LED spatial non-uniformity (7.46%, Table 4) to the uncertainty in $\Delta n_{H}$ was also evaluated. Because the dark measurement was performed without illumination, the spatial irradiance variation affected only $n_{H,\mathrm{light}}$, contributing $u{\left(\Delta n_{H}\right)}_{\mathrm{spatial}}\approx 0.0746\;n_{H,\mathrm{light}}=3.2\times 10^{13}\;{\mathrm{cm}}^{-3}$ for wafer 1. The complete combined standard uncertainty of the apparent Hall-derived carrier-density change, including both the instrumental RSS budget and the illumination-condition (spatial) contribution, was therefore

(11) $$u_{c}\left(\Delta n_{H}\right)=\sqrt{\;u_{c}^{2}\left(n_{H,\mathrm{dark}}\right)+u_{c}^{2}\left(n_{H,\mathrm{light}}\right)+u_{\mathrm{spatial}}^{2}\left(\Delta n_{H}\right)\;},\;u_{\mathrm{spatial}}\left(\Delta n_{H}\right)=s_{\mathrm{LED}}\;n_{H,\mathrm{light}},$$

where $s_{\mathrm{LED}}=0.0746$ is the measured LED spatial non-uniformity (Table 4) and the first two terms reproduce the instrumental RSS budget of Equation (10). Combining this with the instrumental RSS contribution ($u{\left(\Delta n_{H}\right)}_{\mathrm{instr}}\approx 1.6\times 10^{12}\;{\mathrm{cm}}^{-3}$) gave a total $u_{c}\left(\Delta n_{H}\right)\approx 3.2\times 10^{13}\;{\mathrm{cm}}^{-3}$ and a correspondingly reduced ${\mathrm{SNR}}_{\Delta n}\approx 4.3$ for wafer 1. Although this was substantially lower than the instrumental-only estimate of ≈88, wafer 1 remained classified as resolved (${\mathrm{SNR}}_{\Delta n}>3$) even when the full spatial non-uniformity was included. For wafer 2, the spatial contribution did not change the below-threshold classification. The spatial non-uniformity therefore did not alter the detection classification of either wafer; it is reported here as a complete systematic budget entry.

Thus, wafer 1 was classified as resolved, whereas wafer 2 lay below the detection threshold and was retained only as a nominal below-threshold estimate for trend-level discussion.

For wafer 1, the extracted injection ratio was $\Delta n_{H}/N_{D}\approx 0.48$, indicating a light-induced Hall-derived variation that was significantly larger than the combined instrumental uncertainty. According to the ${\mathrm{SNR}}_{\Delta n}$ criterion, this response was classified as resolved. However, because this ratio approaches moderate injection conditions, the Hall-derived carrier density variation should be interpreted as an apparent differential metric rather than a strict low-injection excess-carrier density [1,32].

In contrast, wafer 2 exhibited a much smaller injection ratio of $\Delta n_{H}/N_{D}\approx 9.5\times 10^{-5}$. Under the quantified instrumental RSS criterion, this differential signal lay below the detection threshold. Therefore, the observed photoconductive response for this heavily doped wafer should be interpreted as a nominal below-threshold estimate retained only for trend-level discussion, rather than as a fully resolved quantitative photoconductive variation.

These results emphasize the intrinsic sensitivity limitations of the simplified steady-state Hall-based method under low-injection conditions, particularly in highly doped samples where the equilibrium carrier density is large and the relative illumination-induced variation becomes extremely small [1,4]. Under such conditions, even small Hall-voltage offsets or measurement dispersion can become comparable to the signal of interest [20,21].

Improving detectability in this regime would require enhanced signal-to-noise performance, potentially achieved through higher magnetic field strength, increased Hall voltage resolution, longer integration times, improved shielding, or signal averaging strategies. Alternatively, the use of modulated illumination combined with lock-in detection could significantly improve sensitivity, although at the expense of experimental simplicity [18,28].

### 2.5. Hall-Factor Sensitivity Analysis

The nominal Hall-derived carrier densities reported in this work were calculated using $r_{H}=1$ as the reference assumption. However, the Hall factor in doped silicon may deviate from unity depending on dopant concentration, carrier scattering mechanisms, and temperature. Therefore, the effect of plausible variations in $r_{H}$ was evaluated as a sensitivity analysis rather than as an independent correction, since $r_{H}$ was not directly measured for the present wafers.

For a single majority-carrier semiconductor, the magnitude of the Hall coefficient is given by

(12) $$|R_{H}|=\frac{r_{H}}{qn}.$$

Thus, a carrier density corrected for an assumed Hall factor can be expressed as

(13) $$n_{H}\left(r_{H}\right)=r_{H}n_{H}^{\left(1\right)},$$

where $n_{H}^{\left(1\right)}$ is the nominal Hall-derived carrier density obtained with $r_{H}=1$. If the same effective Hall factor is assumed for the dark and illuminated states, the corresponding apparent carrier-density change also scales linearly:

(14) $$\Delta n_{H}\left(r_{H}\right)=r_{H}\Delta n_{H}^{\left(1\right)}.$$

For the fixed Hall-mobility formulation, keeping the measured dark Hall mobility unchanged, the Hall-based photoconductive estimator becomes:

(15) $$\Delta \sigma_{n,H}\left(r_{H}\right)=r_{H}\Delta \sigma_{n,H}^{\left(1\right)}.$$

A conservative interval of $r_{H}=0.9$–1.3 was considered, consistent with reported Hall scattering factors for phosphorus-doped silicon over broad dopant-density ranges [33]. For the specific doping of wafer 1 ($N_{D}\approx 2.90\times 10^{14}\;{\mathrm{cm}}^{-3}$), the del Alamo and Swanson data indicate a most-probable Hall factor in the narrower range $r_{H}\approx 1.10$–1.15, where lattice scattering is dominant and ionized-impurity scattering is not yet significant. The wider 0.9–1.3 interval is retained in Table 5 for conservatism, but the physically expected range for this doping implies that the most likely $\Delta \sigma_{n,H}$ values for wafer 1 lie between 32.6 and $34.1\;\mathrm{mS}\;{\mathrm{cm}}^{-1}$ (scaling the $r_{H}=1$ result by 1.10–1.15), rather than spanning the full tabulated range. This physically motivated range reduces the effective $r_{H}$ sensitivity band from the conservative −10% to +30% of Table 5 to approximately +10% to +15% relative to the nominal $r_{H}=1$ result, which is the range most relevant for quantitative interpretation of wafer 1. The resulting sensitivity values are summarized in Table 5. For $N_{D}\approx 2.9\times 10^{14}\;{\mathrm{cm}}^{-3}$, the del Alamo–Swanson data place the scattering factor at the upper end of the 1.10–1.15 interval, so the range $r_{H}\approx 1.10$–1.15 is adopted here as the most representative for this wafer.

The assumption of equal Hall factors in dark and illuminated states is discussed in the text; the single-carrier $r_{H}\left(n\right)$ variation across the dark-to-illuminated density range of wafer 1 is less than 3% (see Section 2.5), while the dominant illumination-induced Hall-factor effect arises from mixed conduction and is bounded in the same section.

The sensitivity analysis shows that the absolute Hall-derived carrier densities scale directly with the assumed Hall factor. Within the considered interval, $\Delta n_{H}$ and the fixed-Hall-mobility estimate of $\Delta \sigma_{n,H}$ vary from −10% to +30% relative to the nominal $r_{H}=1$ values. This variation affects the absolute numerical values and injection ratios, but it does not alter the main experimental conclusion: wafer 1 exhibits a clearly resolved Hall-derived response, whereas wafer 2 remains a nominal below-threshold differential signal.

A possible illumination-induced change in the effective Hall factor cannot be excluded. If $r_{H,\mathrm{light}}\neq r_{H,\mathrm{dark}}$, part of the apparent $\Delta n_{H}$ may arise from a change in the Hall coefficient rather than from a true carrier-density variation. This effect is expected to be particularly critical for wafer 2, where the differential signal is below the detection threshold. Therefore, the wafer 2 response is not interpreted quantitatively. A further, and for wafer 1 dominant, illumination-induced Hall-factor effect arises from mixed conduction. Because illumination generates Δn=Δp, the illuminated state is a two-carrier conductor with Hall coefficient $R_{H}=\left(p\mu_{p}^{2}-n\mu_{n}^{2}\right)/\left[q{\left(n\mu_{n}+p\mu_{p}\right)}^{2}\right]$. The hole term lowers $|R_{H}|$, so the single-carrier inversion used here overestimates the true excess electron density. Evaluating this expression for wafer 1 with $\mu_{p}\approx 450\;{\mathrm{cm}}^{2}\;V^{-1}\;s^{-1}$ indicates that a substantial fraction (of order half) of the apparent $\Delta n_{H}$ may arise from the reduced two-carrier Hall coefficient rather than from net majority-carrier generation. This is an order-of-magnitude bound on the interpretation of $\Delta n_{H}$, not a calibrated correction, because $\mu_{p}$, $r_{H,\mathrm{light}}$, and the effective injection level were not independently determined here. For wafer 2, the ratio $p/n\approx 10^{-4}$ renders this correction negligible. The change in the single-carrier scattering factor $r_{H}\left(n\right)$ across the dark-to-illuminated density range is only a few percent: using the phosphorus-doped silicon data of [33], $r_{H}$ varies by less than 3% over the density interval from $n_{H,\mathrm{dark}}=2.93\times 10^{14}\;{\mathrm{cm}}^{-3}$ to $n_{H,\mathrm{light}}=4.31\times 10^{14}\;{\mathrm{cm}}^{-3}$ for wafer 1, well within the $r_{H}=0.9$–1.3 sensitivity range already considered in Table 5. This reinforces the interpretation of $\Delta n_{H}$ as an empirical Hall-derived indicator.

It should also be noted that if the Hall mobility were simultaneously converted to drift mobility using $\mu_{n}=\mu_{H}/r_{H}$, then the product $q\mu_{n}\Delta n_{H}$ would remain unchanged for a common Hall factor in the dark and illuminated states. Therefore, the Hall-factor sensitivity mainly affects the interpretation of absolute Hall-derived carrier densities, while its impact on the conductivity estimator depends on whether the mobility is treated as the measured Hall mobility or as a corrected drift mobility.

## 3. Results

### 3.1. Hall-Derived Carrier Density Under Dark and Illuminated Conditions

**Figure 5:**
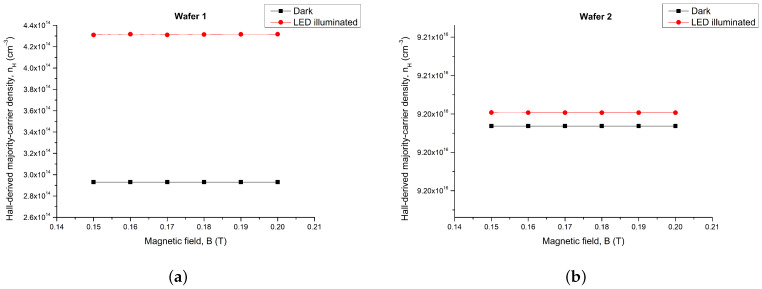
Hall-derived carrier density versus magnetic field under dark and illuminated conditions for (**a**) wafer 1 and (**b**) wafer 2.

The Hall-derived majority-carrier density $n_{H}$ measured under dark and illuminated conditions is presented in Figure 5 as a function of the applied magnetic field for both silicon wafers. The extracted values correspond to electrically measured carrier densities obtained from the Hall coefficient and therefore represent effective Hall-derived concentrations rather than exact chemical dopant densities [4,5,34]. Throughout the Results and Discussion, $\Delta n_{H}$ is treated as an empirical Hall-derived differential *indicator* of the illumination response, not as a measurement of the excess carrier density Δn; as quantified by the mixed-conduction estimate in the Hall-factor sensitivity analysis, the two differ by a non-negligible factor for the lightly doped wafer.

For wafer 1, the carrier density increased from $n_{H,\mathrm{dark}}=2.93\times 10^{14}\;{\mathrm{cm}}^{-3}$ under dark conditions to $n_{H,\mathrm{light}}=4.31\times 10^{14}\;{\mathrm{cm}}^{-3}$ under illumination at $1.81\;\mathrm{mW}\;{\mathrm{cm}}^{-2}$. Using the unrounded Hall-derived values, this gave an apparent carrier-density change of $\Delta n_{H}=n_{H,\mathrm{light}}-n_{H,\mathrm{dark}}\approx 1.38\times 10^{14}\;{\mathrm{cm}}^{-3}$. Using the resistivity–mobility consistency estimate $N_{D}\approx 2.90\times 10^{14}\;{\mathrm{cm}}^{-3}$, the corresponding apparent injection ratio was $\Delta n_{H}/N_{D}\approx 0.48\pm 0.03$, indicating that the wafer operated within a moderate (transitional) injection regime rather than a strict low-injection regime [4,32].

In contrast, wafer 2 showed only a nominal below-threshold Hall-derived differential signal. Because the dark and illuminated Hall-derived carrier densities differed only in the fourth significant digit, the apparent carrier-density change was calculated from the unrounded values, yielding $\Delta n_{H}\approx 9.13\times 10^{12}\;{\mathrm{cm}}^{-3}$. With an estimated dopant density of $N_{D}\approx 9.66\times 10^{16}\;{\mathrm{cm}}^{-3}$, this corresponded to an apparent injection ratio of $\Delta n_{H}/N_{D}\approx 9.5\times 10^{-5}$. Since this differential signal lies below the detection-threshold criterion, it should be interpreted as a nominal below-threshold response, consistent with low-level injection in heavily doped silicon, rather than as a fully resolved quantitative excess-carrier measurement [4,27,32].

For both wafers, the Hall-derived carrier density showed negligible dependence on the magnetic field within the explored range (150–200mT), indicating stable Hall extraction and the absence of magnetic-field-induced artifacts. The short-term repeatability of the extracted values supported the use of the steady-state Hall configuration for comparative analysis [20,21]. The measured light-induced carrier-density variation constituted the basis for the estimation of the Hall-based photoconductive response presented in subsequent sections. Accordingly, every quantity derived from $\Delta n_{H}$ and $\Delta \sigma_{n,H}$ in the following sections must be read strictly as a comparative Hall-derived indicator—valid for relative, wafer-to-wafer screening under identical measurement conditions—and not as an absolute photoconductivity or excess-carrier-density value.

### 3.2. Hall Mobility and Constant-Mobility Approximation

**Figure 6:**
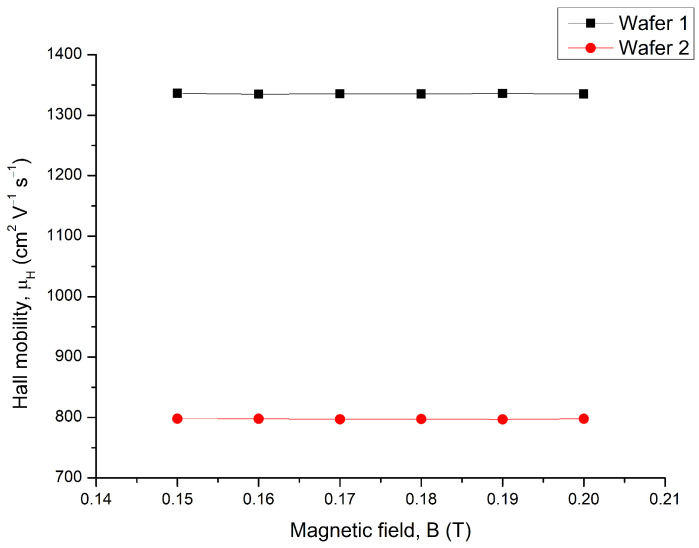
Hall mobility versus magnetic field under dark conditions for wafer 1 and wafer 2. Error bars denote the sample standard deviation over the field sweep (Table 2); they are smaller than the symbol size due to the low dispersion of the field-sweep data (CV<0.13%). The illuminated Hall mobility, computed from $R_{H,\mathrm{light}}$ and $\rho_{\mathrm{light}}$, overlaps $\mu_{H,\mathrm{dark}}$ within the field-sweep uncertainty for both wafers, confirming that no illumination-induced mobility change is resolved within the present experimental dispersion. Wafer 1 (■, upper series) and wafer 2 (•, lower series) are shown on the same axis.

The Hall mobility measured under dark conditions is presented in Figure 6 for both wafers as a function of the applied magnetic field. Wafer 1 exhibited an average Hall mobility of $\mu_{H}=1335.48\pm 1.64\;{\mathrm{cm}}^{2}\;V^{-1}\;s^{-1}$, whereas wafer 2 showed a lower value of $\mu_{H}=797.44\pm 0.98\;{\mathrm{cm}}^{2}\;V^{-1}\;s^{-1}$ (field-sweep sample standard deviations, Table 2). This reduction in mobility for the heavily doped sample is consistent with the expected degradation caused by ionized-impurity scattering at high dopant concentrations [5,27].

Within the experimental dispersion of the present setup, no systematic mobility variation was resolved under illumination. Therefore, the dark Hall mobility $\mu_{H,\mathrm{dark}}$ was adopted as a first-order approximation for the estimation of the photoconductive response. This assumption was well justified for wafer 2, since its injection ratio remained extremely low, implying negligible perturbation of transport parameters. For wafer 1, the injection level reached a moderate (transitional) regime, where slight mobility deviations could potentially occur due to carrier–carrier interactions or recombination-induced transport changes. However, the experimental data did not show variations beyond measurement dispersion, supporting the use of a constant-mobility approximation [1,5]. At the wafer 1 injection ratio $\Delta n_{H}/N_{D}\approx 0.48$, the constant-mobility approximation nonetheless remained justified: the majority-carrier mobility depended on carrier density only through carrier–carrier scattering, which was negligible below *n*∼$10^{17}\;{\mathrm{cm}}^{-3}$. The Arora model predicts a mobility change of order 1% across the dark-to-illuminated density range, far smaller than the 11.5% cycling dispersion of Table 3. At this injection ratio it was therefore the neglect of minority carriers, not the constant-mobility assumption, that limited the majority-carrier estimator; that contribution was bounded by the bipolar estimate $\Delta \sigma_{\mathrm{bip}}$ of the following subsection, which reduced the deviation from the Macdonald–Cuevas model from 44.3% to 24.4%. Computing the illuminated Hall mobility from the measured $R_{H,\mathrm{light}}$ and $\rho_{\mathrm{light}}$ yielded a value that overlapped $\mu_{H,\mathrm{dark}}$ within the field-sweep uncertainty of Table 2, confirming that no illumination-induced mobility change was resolved [35].

In addition, the weak dependence of $\mu_{H}$ on magnetic field for both wafers suggests stable Hall extraction and supports the absence of significant field-dependent measurement artifacts within the explored range. To evaluate the impact of the constant-mobility assumption, a sensitivity analysis was conducted by allowing $\mu_{n}$ to vary within its propagated uncertainty range. The resulting variation in the Hall-based photoconductive estimator was found to be smaller than the dominant uncertainty contributions associated with the carrier-density extraction, indicating that the approximation does not significantly affect the conclusions of the present comparative study [29].

### 3.3. Estimation of the Hall-Derived Majority-Carrier Photoconductive Response

The Hall-based majority-carrier photoconductive response was estimated from the apparent increase in Hall-derived carrier density, $\Delta n_{H}$, using the dark Hall mobility, $\mu_{H,\mathrm{dark}}$, as a reference mobility according to Equation (3). The resulting dark-referenced Hall-derived estimator, denoted as $\Delta \sigma_{n,H}$, was obtained for both wafers under white LED illumination at an irradiance of $1.81\;\mathrm{mW}\;{\mathrm{cm}}^{-2}$.

**Figure 7:**
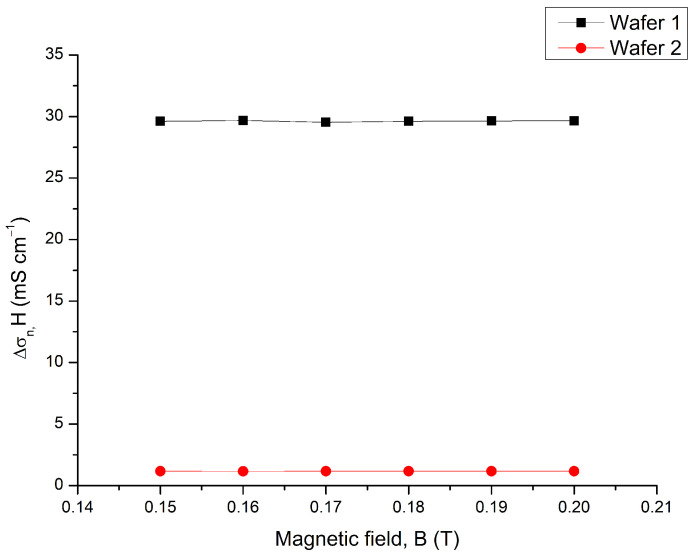
Hall-based photoconductive response versus magnetic field under LED illumination ($1.81\;\mathrm{mW}\;{\mathrm{cm}}^{-2}$) for wafer 1 and wafer 2.

For wafer 1, the extracted value was $\Delta \sigma_{n,H}\approx 2.96\times 10^{-2}\;S\;{\mathrm{cm}}^{-1}$ ($29.6\;\mathrm{mS}\;{\mathrm{cm}}^{-1}$), indicating a clearly resolved Hall-derived response. In contrast, wafer 2 yielded a nominal below-threshold estimate of $\Delta \sigma_{n,H}\approx 1.17\times 10^{-3}\;S\;{\mathrm{cm}}^{-1}$ ($1.17\;\mathrm{mS}\;{\mathrm{cm}}^{-1}$), as shown in Figure 7. This value should be interpreted as a nominal below-threshold response, retained only for trend-level comparison rather than as a fully resolved quantitative photoconductive variation.

To evaluate the potential contribution of minority carriers, a bipolar photoconductivity estimate was also calculated assuming Δn≈Δp and using representative hole mobility values at room temperature. This yielded $\Delta \sigma_{\mathrm{bip}}\approx 4.00\times 10^{-2}\;S\;{\mathrm{cm}}^{-1}$ for wafer 1 and $\Delta \sigma_{\mathrm{bip}}\approx 1.48\times 10^{-3}\;S\;{\mathrm{cm}}^{-1}$ for wafer 2. The comparison between $\Delta \sigma_{n,H}$ and $\Delta \sigma_{\mathrm{bip}}$ indicated that the hole contribution was non-negligible, particularly for the lightly doped wafer: $\Delta \sigma_{\mathrm{bip}}$ exceeds $\Delta \sigma_{n,H}$ by approximately 35% for wafer 1 (40.0 vs. $29.6\;\mathrm{mS}\;{\mathrm{cm}}^{-1}$), which should be regarded as a systematic underestimation inherent to the majority-carrier-only Hall formulation under moderate injection. It was therefore the omission of minority-carrier contributions, rather than the constant-mobility approximation, that constituted the dominant systematic limitation of the majority-carrier Hall estimator at the injection ratio $\Delta n_{H}/N_{D}\approx 0.48$ of wafer 1. The majority-carrier component nonetheless remained dominant. Because $\Delta \sigma_{\mathrm{bip}}$ was evaluated from the same apparent $\Delta n_{H}$, it was an upper-bound sensitivity estimate of the hole drift contribution rather than a calibrated value. It was therefore not combined with the mixed-conduction overestimate discussed in the Hall-factor sensitivity analysis: the two are independent order-of-magnitude bounds—one on the apparent Hall density and one on the conductivity—and are not additive. Consequently, any agreement between $\Delta \sigma_{\mathrm{bip}}$ and the Macdonald–Cuevas trend is regarded as qualitative only, since a quantitative reconciliation would require independent determination of $\mu_{p}$, $r_{H,\mathrm{light}}$, and the true excess-carrier density.

**Figure 8:**
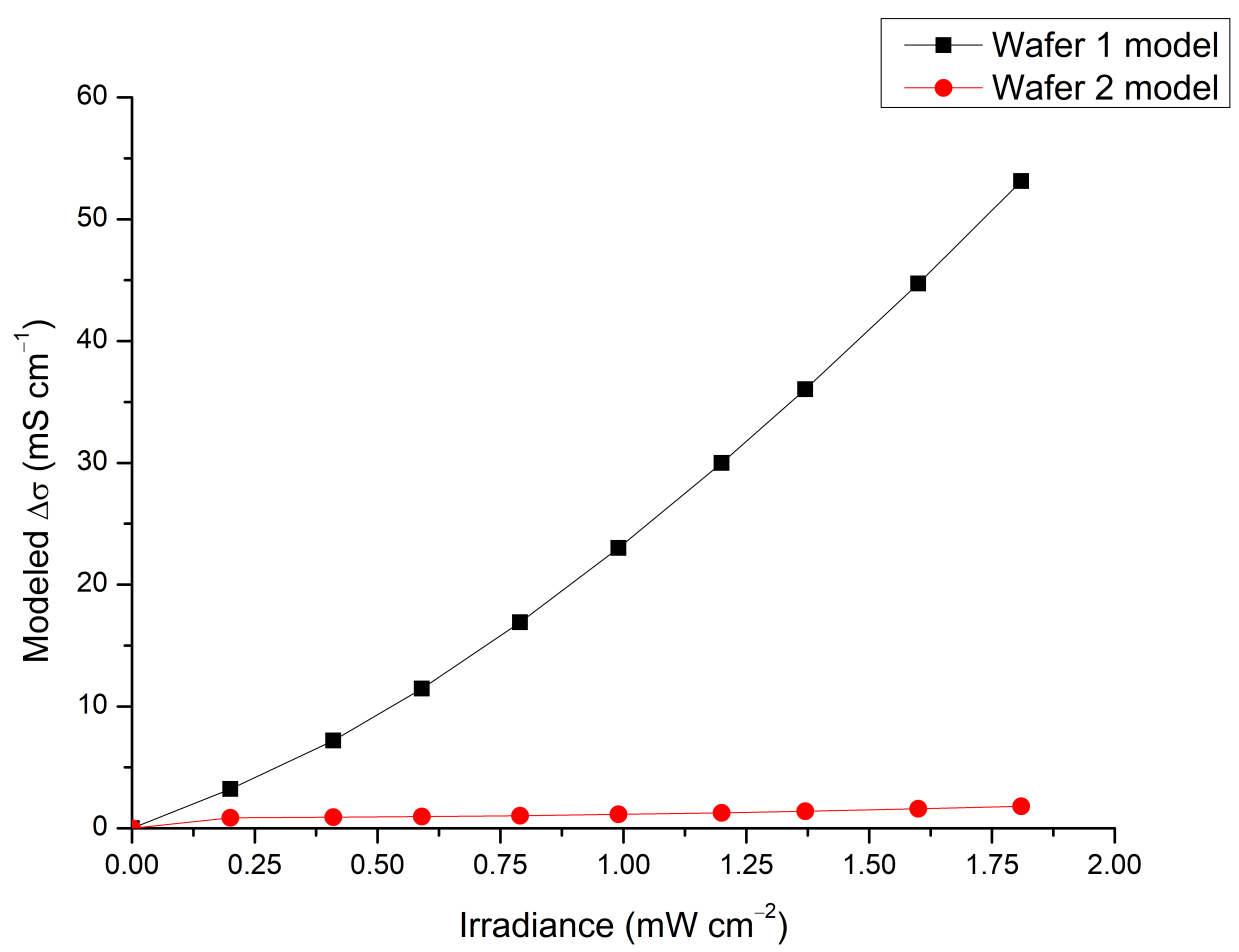
Macdonald–Cuevas model photoconductive response versus irradiance over the experimental range.

The experimental results were compared with the Macdonald–Cuevas model [30,31], which accounts for recombination and carrier trapping/de-trapping effects in crystalline silicon. The model was evaluated using dopant densities of $N_{D}=2.90\times 10^{14}\;{\mathrm{cm}}^{-3}$ for wafer 1 and $N_{D}=9.66\times 10^{16}\;{\mathrm{cm}}^{-3}$ for wafer 2, over the irradiance range from 0 to $1.81\;\mathrm{mW}\;{\mathrm{cm}}^{-2}$, as shown in Figure 8. The comparison suggests that the Hall-based estimator followed the expected qualitative dependence of the photoconductive response on both doping concentration and illumination intensity. However, systematic deviations in absolute magnitude were observed, which can be attributed to the simplified treatment of minority-carrier contributions, transport effects, and spatially non-uniform optical generation inherent to the steady-state Hall approach.

### 3.4. Repeatability and Short-Term Stability Indicators

Repeatability and stability indicators were obtained from the magnetic-field sweep and current-dependent measurements. The magnetic-field sweep was used to evaluate the dispersion of $\mu_{H}$, $\Delta n_{H}$, and $\Delta \sigma_{n,H}$ over six field values, whereas the current sweep was used to evaluate the dispersion of the Van der Pauw resistivity over ten current values. These statistics represent short-term measurement stability and sweep-to-sweep dispersion, not independent remounting reproducibility. The repeatability and stability statistics summarized in Table 2 show low dispersion, with CV values below 0.23% for the Hall-derived quantities obtained from the magnetic-field sweep.

The statistics reported in Table 2 correspond to magnetic-field and current-sweep stability. To quantify the practical reproducibility of the complete two-state protocol, additional dark/light cycling measurements were performed using three independent mountings and five cycles per mounting for each wafer. For each cycle, $\Delta n_{H}$ was calculated from the difference between the illuminated and dark Hall-derived carrier densities, and $\Delta \sigma_{n,H}$ was obtained using the dark Hall mobility. The resulting remounting and cycling statistics are summarized in Table 3; SD denotes the sample standard deviation over the indicated cycles. The 15-cycle statistics preserve the same mean values used in the main Hall-derived estimator while adding an experimental measure of mounting and cycling dispersion. Wafer 1 remained clearly resolved under the present illumination conditions. Wafer 2 showed repeatable nominal values across the cycling protocol, but the differential Hall signal remained below the detection-limit criterion and was therefore retained only as a trend-level estimate. The individual dark/light cycling values are provided in Supplementary Table S1.

### 3.5. Irradiance Dependence and Summary Comparison of the Hall-Derived Estimator

The irradiance dependence of the Hall-derived majority-carrier photoconductive estimator was evaluated using the dark/illuminated differential protocol described above. Measurements were conducted at ten irradiance levels using a fixed Hall current of 50μA and a constant magnetic field of approximately 200mT. The resulting trend was compared with the Macdonald–Cuevas model as a qualitative physical-consistency benchmark rather than as a calibrated reference measurement.

At the maximum experimental irradiance of $1.81\;\mathrm{mW}\;{\mathrm{cm}}^{-2}$, wafer 1 ($N_{D}\approx 2.90\times 10^{14}\;{\mathrm{cm}}^{-3}$, ρ≈16.15Ωcm) exhibited an apparent Hall-derived carrier-density change of $\Delta n_{H}\approx 1.38\times 10^{14}\;{\mathrm{cm}}^{-3}$, corresponding to a Hall-derived majority-carrier photoconductive response of $\Delta \sigma_{n,H}\approx 29.6\;\mathrm{mS}\;{\mathrm{cm}}^{-1}$. When the bipolar contribution was estimated by including an approximate hole contribution, the corresponding sensitivity estimate increased to $\Delta \sigma_{\mathrm{bip}}\approx 40.0\;\mathrm{mS}\;{\mathrm{cm}}^{-1}$.

In contrast, wafer 2 ($N_{D}\approx 9.66\times 10^{16}\;{\mathrm{cm}}^{-3}$, ρ≈0.081Ωcm) yielded a nominal below-threshold apparent carrier-density change of $\Delta n_{H}\approx 9.13\times 10^{12}\;{\mathrm{cm}}^{-3}$, corresponding to $\Delta \sigma_{n,H}\approx 1.17\;\mathrm{mS}\;{\mathrm{cm}}^{-1}$. The corresponding bipolar sensitivity estimate was $\Delta \sigma_{\mathrm{bip}}\approx 1.48\;\mathrm{mS}\;{\mathrm{cm}}^{-1}$. These values indicate a substantially weaker apparent response for the heavily doped wafer; however, no quantitative wafer-to-wafer response ratio was claimed because the wafer 2 differential Hall signal lay below the instrumental detection threshold according to the ${\mathrm{SNR}}_{\Delta n}$ criterion.

**Figure 9:**
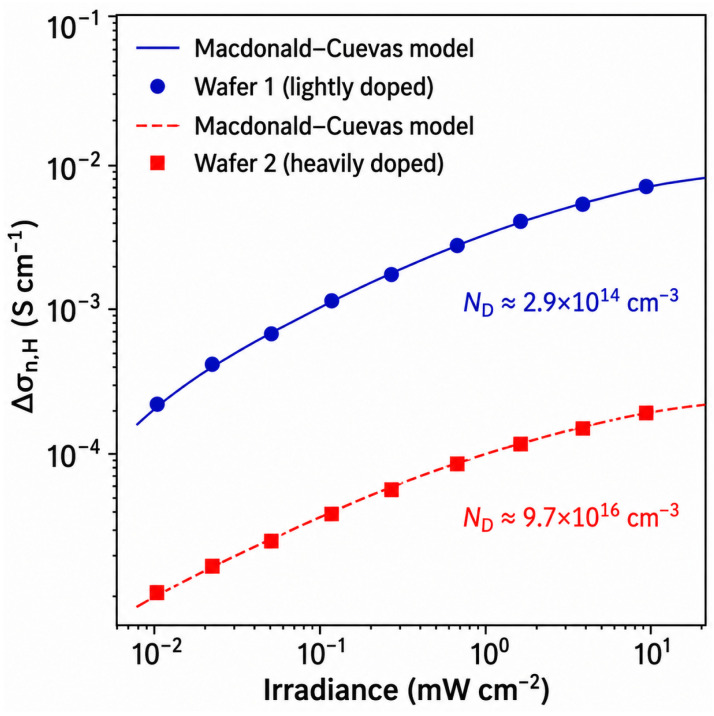
Summary comparison of the Hall-derived majority-carrier photoconductive response, $\Delta \sigma_{n,H}$, as a function of irradiance for the lightly doped and heavily doped *n*-type silicon wafers. Symbols represent the Hall-derived dark/illuminated differential estimator, whereas lines represent the corresponding Macdonald–Cuevas model trends. The lightly doped wafer shows a clearly resolved monotonic response, while the heavily doped wafer is retained only as a nominal below-threshold trend-level indication.

As shown in Figure 9 and summarized in Table 6, the Hall-derived estimator captured the expected monotonic increase in photoconductive response for wafer 1. For wafer 2, the same behavior was retained only as a nominal trend-level indication because the differential Hall signal remained below the detection-threshold criterion. The comparison with the Macdonald–Cuevas model supported the qualitative doping-dependent behavior of the measured response: the lightly doped wafer exhibited a larger resolved response, whereas the heavily doped wafer showed a much smaller apparent response due to its large equilibrium majority-carrier density and the correspondingly small relative light-induced variation.

The absolute Hall-derived values were lower than the Macdonald–Cuevas model predictions. At $1.81\;\mathrm{mW}\;{\mathrm{cm}}^{-2}$, the relative deviation of the majority-carrier Hall-derived estimate was 44.3% for wafer 1. When the bipolar sensitivity estimate was considered, this deviation decreased to 24.4%, indicating that part of the discrepancy arose from the omission of minority-carrier contributions in the majority-carrier Hall formulation. For wafer 2, the apparent deviation was not interpreted quantitatively because the underlying differential Hall signal lay below the instrumental detection threshold.

The remaining differences can be attributed to effects not explicitly included in the simplified estimator, including trapping and de-trapping mechanisms, surface recombination, non-uniform depth-dependent optical generation under broadband illumination, parasitic photovoltage contributions, and the use of the dark Hall mobility as a reference mobility for the illuminated-state estimation. Therefore, Figure 9 should be interpreted as a trend-comparison and physical-consistency summary, not as a calibrated validation of the Hall-derived estimator against the Macdonald–Cuevas model.

### 3.6. Longitudinal Van der Pauw Consistency Check

As an internal experimental consistency check, the longitudinal conductivity change was independently estimated from the Van der Pauw resistivity measured under dark and illuminated conditions. The corresponding conductivity values were calculated as

(16) $$\sigma_{\mathrm{dark}}^{\mathrm{VdP}}=\frac{1}{\rho_{\mathrm{dark}}}$$

(17) $$\sigma_{\mathrm{light}}^{\mathrm{VdP}}=\frac{1}{\rho_{\mathrm{light}}}$$

and the longitudinal photoconductive response was obtained as

(18) $$\Delta \sigma_{\mathrm{VdP}}=\sigma_{\mathrm{light}}^{\mathrm{VdP}}-\sigma_{\mathrm{dark}}^{\mathrm{VdP}}=\frac{1}{\rho_{\mathrm{light}}}-\frac{1}{\rho_{\mathrm{dark}}}.$$

This quantity represents the total longitudinal conductivity change measured by the Van der Pauw configuration and is therefore not equivalent to the Hall-derived majority-carrier estimator $\Delta \sigma_{n,H}$. Nevertheless, agreement in sign, relative trend, and order of magnitude between $\Delta \sigma_{\mathrm{VdP}}$ and $\Delta \sigma_{n,H}$ provides an internal consistency check for the illumination-induced electrical response.

For wafer 1, the longitudinal Van der Pauw response and the Hall-derived estimator agreed closely (Table 7), supporting the internal consistency of the illumination-induced electrical response. For wafer 2, the mean values also agreed in sign and magnitude; however, the response was extremely small relative to the dark conductivity and should therefore be interpreted as a below-threshold internal consistency comparison rather than as a resolved quantitative response. Because $\Delta \sigma_{\mathrm{VdP}}$ is obtained from longitudinal resistivity whereas $\Delta \sigma_{n,H}$ is derived from the Hall coefficient and dark Hall mobility, exact numerical agreement is not generally expected. Differences can arise from minority-carrier contributions, illumination-induced mobility changes, contact effects, non-uniform photogeneration, and residual systematic offsets. Therefore, this comparison is used only as an internal consistency check and not as a calibrated reference comparison against a photoconductance technique.

## 4. Discussion

### 4.1. Physical Interpretation

The observed variation in the Hall-based photoconductive response between the two *n*-type crystalline silicon wafers was consistent with established carrier generation, recombination, and transport mechanisms in doped silicon [1,4,36]. Under steady-state illumination, the photoconductive response depends on the balance between optical carrier generation and recombination, as well as on the mobility of the charge carriers contributing to conduction [6,28].

The lightly doped wafer (wafer 1) exhibited a significantly larger increase in Hall-derived carrier density and photoconductivity, indicating a greater ability to sustain excess carrier populations under illumination. This behavior was expected because lower dopant concentrations generally reduce recombination rates and allow longer effective carrier lifetimes, enabling a larger steady-state accumulation of photogenerated carriers [1,32].

In contrast, the heavily doped wafer (wafer 2) showed only a nominal below-threshold Hall-derived response. This response was consistent with the expected reduction in excess-carrier accumulation at high doping; however, it should not be used for quantitative mechanistic attribution without improved signal-to-noise performance or independent benchmarking. Mechanisms such as Auger recombination, impurity-related recombination, and mobility degradation may contribute to this trend, but they cannot be independently resolved using the present two-state Hall-derived estimator alone [30,31,37,38,39,40].

Furthermore, the mobility measured for wafer 2 was substantially lower than that of wafer 1, consistent with the increased ionized-impurity scattering associated with higher dopant densities [5,27]. Since photoconductivity depends directly on both carrier concentration and mobility, the combined effect of reduced mobility and reduced excess carrier accumulation was consistent with the much smaller nominal response observed for wafer 2 [1,5].

Overall, the resolved response of wafer 1 and the nominal below-threshold response of wafer 2 were consistent with classical semiconductor transport theory and previous studies of doped silicon, which have consistently reported a decreasing photoconductive response with increasing doping concentration due to the combined effects of enhanced recombination, impurity scattering, and defect-related carrier losses [4,27,36,37,41].

### 4.2. Internal Consistency and Scope of Applicability

The stability and internal consistency of the experimental system were assessed by sweeping the magnetic field between 150 and 200mT in 10mT increments for both wafers using the classical Hall configuration (Figure 5). The repeatability and stability statistics summarized in Table 2 show low dispersion for the Hall-derived quantities obtained from the magnetic-field sweep, with CV values below 0.23%. The additional three-mounting/five-cycle dark-light dataset in Table 3 quantifies the practical repeatability of the full two-state protocol, yielding total CV values of 16.50% and 13.50% for $\Delta n_{H}$ for wafers 1 and 2, respectively, and 11.50% and 12.00% for $\Delta \sigma_{n,H}$. These results support the short-term stability of the measurement procedure and indicate that the Hall extraction remains stable within the selected magnetic-field range, while also showing that remounting and dark/light cycling introduce a larger but measurable dispersion component [20,21].

The proposed approach provides a low-complexity Hall-based estimator of the photoconductive response, derived from the illumination-induced variation in the Hall carrier density. Its applicability is primarily constrained by the assumptions inherent to majority-carrier-dominated transport and the constant mobility approximation [1,4,5]. Consequently, the method is most reliable under low-to-moderate injection conditions, where conductivity remains largely governed by majority carriers and illumination-induced mobility changes are limited [1,32].

An additional limitation arises from the absence of a depth-resolved optical generation model. Under broadband white LED illumination, the photogeneration profile within crystalline silicon is inherently non-uniform due to the strong wavelength dependence of the absorption coefficient. Short-wavelength components are absorbed near the surface, while longer wavelengths penetrate deeper into the bulk [23,24]. As a result, the extracted Hall-derived carrier density should be interpreted as an effective volume-averaged quantity, influenced by both bulk recombination and surface recombination processes [1,6].

The proposed method is therefore best suited for crystalline materials with moderate defect density when the primary objective is to capture relative trends rather than extract fully resolved carrier transport parameters [28]. Within this scope, the Hall-based estimator showed a clearly resolved monotonic irradiance dependence for wafer 1 and only a nominal below-threshold trend for wafer 2, while preserving the qualitative doping dependence described by the Macdonald–Cuevas model [30,31].

Overall, these considerations define the practical scope of the method as an accessible tool for comparative photoconductive screening, particularly in experimental environments where simplicity and low instrumentation cost are prioritized over complete physical resolution [11,18].

### 4.3. Scope and Limitations of the Hall-Derived Estimator

The proposed steady-state Hall-based method presents several inherent limitations associated with both its simplified analytical formulation and its experimental implementation.

A primary limitation is the systematic deviation observed between the Hall-based photoconductivity estimator and the Macdonald–Cuevas model predictions (Figure 9). The Hall-based approach primarily reflects the response of majority carriers extracted from the measured Hall coefficient, whereas the model includes additional physical contributions such as minority-carrier transport, trapping and de-trapping effects, surface recombination, and injection-dependent recombination dynamics [30,31]. Therefore, the observed differences in absolute magnitude are expected and reflect the reduced physical completeness of the Hall-based estimator rather than an experimental calibration error [8].

The adopted two-state protocol (dark versus continuous illumination) does not provide time resolution and cannot capture transient photoconductivity effects. Consequently, processes such as carrier trapping kinetics, delayed recombination dynamics, or illumination-induced metastable behavior cannot be separated. More advanced techniques, including carrier-resolved photo-Hall or time-dependent photoconductance methods, are required when temporal carrier dynamics must be explicitly resolved [8,11,16,17,18]. Recent broadband time-resolved photoconductance and optical-pump terahertz-probe systems occupy the high-resolution, high-complexity end of this spectrum; the present steady-state estimator is positioned as their accessible, low-cost complement rather than as a competitor.

Another limitation is the assumption of constant majority-carrier mobility under illumination. Although no systematic mobility variation was resolved within the experimental dispersion of the present crystalline silicon wafers, this approximation may not remain valid for materials exhibiting strong carrier–carrier scattering, heating effects, or illumination-induced changes in defect-mediated scattering [5,27]. Under moderate or high injection conditions, mobility variations may become non-negligible, introducing systematic bias in the extracted photoconductive response [1]. On the basis of the ≈35% majority-carrier underestimation observed at $\Delta n_{H}/N_{D}\approx 0.48$, and the approximately linear growth of the hole contribution with injection, the majority-carrier-only estimator is expected to remain within ∼10% of the full bipolar response only for $\Delta n_{H}/N_{D}≲0.05$, i.e., roughly an order of magnitude below the injection level of wafer 1 under the present illumination.

In addition, Hall measurements under illumination may be affected by parasitic photovoltages, contact misalignment, and photo-thermoelectric offsets. While these effects were mitigated through magnetic-field reversal, symmetric Van der Pauw geometry, and electromagnetic shielding, they remain potential sources of systematic uncertainty, particularly when the illumination-induced Hall signal approaches the detection limit [18,20,21].

The extracted carrier density corresponds to a nominal Hall-derived value obtained using $r_{H}=1$ as the reference assumption. The Hall-factor sensitivity analysis shows that plausible variations in $r_{H}$ scale the absolute carrier densities and injection ratios, but they do not modify the qualitative comparison between wafers. Therefore, the reported carrier densities should be interpreted as effective electrical carrier concentrations rather than absolute dopant densities, unless independent calibration is performed [4,5,20].

A further limitation is that the method was demonstrated only at room temperature (300±0.5K). Since carrier mobility, recombination rates, and trap kinetics exhibit strong temperature dependence, extension of the approach to variable-temperature conditions would require additional benchmarking [5,27].

Independent dark/light cycling and sample-remounting reproducibility tests were performed over three mountings with five cycles per mounting, providing a short-term reproducibility estimate for the complete two-state protocol. However, these tests do not constitute a full long-term reproducibility assessment because day-to-day drift, contact aging, repeated re-contacting, and operator-dependent alignment were not separately quantified. These residual contributions are therefore still considered systematic limitations together with illumination alignment and residual parasitic photovoltages.

Finally, the broadband white LED illumination introduces a depth-dependent optical generation profile due to wavelength-dependent absorption in silicon. Consequently, the extracted carrier-density variation represents an effective volume-averaged response rather than a spatially resolved carrier distribution [23,24]. Moreover, no independent experimental benchmark using calibrated QSSPC, SSPC, μ-PCD, or carrier-resolved photo-Hall measurements was performed on the same wafers. Thus, comparison with the Macdonald–Cuevas model should be interpreted as a qualitative physical-consistency benchmark rather than as a calibrated reference comparison [8,11,42,43,44].

### 4.4. Limitations Due to the Absence of Independent Validation

Because no additional measurements or samples were available, the present dataset cannot establish the absolute accuracy of the Hall-derived estimator relative to calibrated photoconductance or carrier-resolved photo-Hall methods. The internal Van der Pauw comparison, magnetic-field stability, Hall-factor sensitivity analysis, and literature comparison support the physical plausibility and trend consistency of the method, but they do not constitute independent experimental validation. Specifically, three quantities cannot be established from the present dataset: (i) the absolute accuracy of the estimator, (ii) whether its response scales correctly with excess carrier density, and (iii) whether the resolved trend originates from photoconductivity or from a Hall-coefficient artifact. Regarding (iii), for wafer 1 the close agreement between the Hall-derived estimator and the independent longitudinal Van der Pauw response ($\Delta \sigma_{n,H}/\Delta \sigma_{\mathrm{VdP}}=1.001$, Table 7) together with ${\mathrm{SNR}}_{\Delta n}\approx 88$ indicates that the resolved response is not dominated by a Hall-coefficient artifact, although it does not constitute calibrated validation.

This limitation is particularly important for the heavily doped wafer, whose light-induced differential signal is below the instrumental detection threshold. Therefore, the wafer 2 values are retained only as nominal indicators of the expected weak response in highly doped silicon. They should not be used to infer quantitative recombination mechanisms, calibrated excess-carrier densities, or an experimentally resolved ratio between the two wafers.

Based on the ${\mathrm{SNR}}_{\Delta n}$ criterion, the minimum conditions for the method to produce a resolved response can be stated explicitly: (i) the injection ratio $\Delta n_{H}/N_{D}$ must exceed $3\sqrt{2}\;u_{c}\left(n_{H}\right)/n_{H}\approx 0.013$ (for the present instrumental RSS budget), which sets a practical doping ceiling of approximately $N_{D}≲10^{15}\;{\mathrm{cm}}^{-3}$ at the present irradiance for crystalline silicon with carrier lifetimes comparable to wafer 1; (ii) the illumination irradiance must be sufficient to generate $|\Delta n_{H}|≳3\;u_{c}\left(\Delta n_{H}\right)$; and (iii) the measurement temperature should be controlled to ±0.5K or better to avoid thermally induced Hall-voltage drift. A simple scaling analysis shows that bringing wafer 2 ($N_{D}\approx 9.66\times 10^{16}\;{\mathrm{cm}}^{-3}$) into the resolved regime under the present instrumental noise floor would require $|\Delta n_{H}|\;≳1.2\;\times \;10^{15}\;{\mathrm{cm}}^{-3}$, which under linear injection scaling corresponds to an irradiance of approximately $230\;\mathrm{mW}\;{\mathrm{cm}}^{-2}$—more than 100 times the present source intensity and physically unrealistic for this type of LED-based setup. This confirms that the method is not practically applicable to silicon wafers with $N_{D}≳10^{16}\;{\mathrm{cm}}^{-3}$ under low-irradiance steady-state conditions, and that resolving such samples would require a fundamentally different approach (e.g., lock-in photoconductance or carrier-resolved photo-Hall).

The present contribution should therefore be read as a proof-of-concept and scope-defining study for a low-complexity Hall-derived screening estimator. A rigorous metrological assessment would require independent QSSPC, SSPC, μ-PCD, WCT, or carrier-resolved photo-Hall measurements on the same samples, together with broader day-to-day remounting, re-contacting, and dark/light cycling statistics.

### 4.5. Scope of the Present Screening System Relative to Established Photoconductance and Photo-Hall Techniques

The Hall-based majority-carrier photoconductive estimates obtained in this work follow the expected qualitative dependence of photoconductive response on doping concentration in crystalline silicon. Under identical illumination conditions, the heavily doped wafer exhibits a much smaller nominal below-threshold response than the lightly doped wafer, consistent with the expected influence of dopant concentration, recombination, and mobility degradation in doped Si [1,27,37].

Nevertheless, the extracted values must be interpreted within the scope of a Hall-derived majority-carrier estimator rather than as fully calibrated photoconductivity measurements. Conventional steady-state photoconductance (SSPC) and quasi-steady-state photoconductance (QSSPC) methods provide calibrated photoconductance and effective lifetime information under controlled illumination and measurement geometries [7,8,9,10]. In contrast, the present method estimates the photoconductive response from the illumination-induced variation of Hall-derived carrier density and does not explicitly resolve minority-carrier contributions, surface recombination, trapping, depth-dependent generation, or injection-dependent transport effects. Therefore, systematic deviations from calibrated photoconductance-based methods are expected, particularly in absolute magnitude [30,31].

Compared with carrier-resolved photo-Hall and light-induced magnetotransport approaches, the present setup offers substantially reduced experimental complexity but also reduced physical completeness. Advanced methods can decouple electron and hole transport parameters under illumination, whereas the simplified steady-state Hall configuration used here provides only a majority-carrier-based estimator suitable for comparative screening [11,18]. Thus, the proposed approach should be considered complementary to SSPC, QSSPC, carrier-resolved photo-Hall, and CLIMAT rather than a substitute for them.

The normalized quantities in Table 8 clarify which aspects can be compared with the literature: dopant concentration, injection ratio, order of magnitude, and response trend. Wafer 2 is therefore retained as a nominal below-threshold case.

Although no independent QSSPC, SSPC, or carrier-resolved photo-Hall measurement was performed on the same wafers, reported studies on crystalline silicon provide a useful literature-based consistency benchmark. Yoon et al. [45] investigated phosphorus-doped *n*-type Cz-Si wafers with $N_{D}\approx 9.2\times 10^{14}\;{\mathrm{cm}}^{-3}$ using QSSPC and RCPCD, a doping level close to wafer 1. Richter et al. [37] reported injection-dependent recombination measurements over a broad crystalline-silicon doping range from approximately $4.6\times 10^{13}$ to $2.5\times 10^{17}\;{\mathrm{cm}}^{-3}$, spanning both wafers studied here. Roller et al. [46] used LED-based QSSPC on photovoltaic-grade silicon wafers, highlighting the influence of spectral content, generation depth, surface recombination, and passivation quality, which is directly relevant to the present white-LED configuration. Table 9 therefore separates quantitative similarity in doping, technique, and injection or illumination regime from limitations in direct comparability of absolute lifetime or photoconductivity values. Because the cited studies used different illumination spectra, passivation conditions, sample geometries, and carrier-resolved models, the comparison is used only for doping-range and injection-regime consistency, not as an absolute calibrated reference comparison for $\Delta \sigma_{n,H}$.

Before turning to the literature-based comparison of Table 9, the methodological scope and principal limitations of the present work are summarized in consolidated form. Although a direct experimental comparison against calibrated QSSPC, μ-PCD, or carrier-resolved photo-Hall measurements would unquestionably strengthen the present work, such a comparison is not a prerequisite for establishing the feasibility of the proposed Hall-derived differential screening methodology. The objective of this study was therefore methodological rather than metrological: to define the operating domain, the sensitivity limits, and the practical applicability of a low-cost Hall-based screening system under controlled steady-state illumination, rather than to certify its absolute accuracy against a reference technique.

Within this objective, the principal limitations can be stated concisely as follows: (i) the absence of independent calibrated photoconductance measurements on the same wafers, so that the absolute accuracy of $\Delta \sigma_{n,H}$ cannot be established; (ii) the majority-carrier approximation, which omits the minority-carrier (hole) contribution under moderate injection and underestimates the bipolar response by approximately 35% for the lightly doped wafer; (iii) the inability to resolve illumination-induced Hall-factor variations, since the experimental quantification of an illumination-dependent $r_{H}$ would require carrier-resolved photo-Hall instrumentation that lies beyond the scope of the present proof-of-concept implementation; and (iv) an applicability that, under the present sub-$\mathrm{mW}\;{\mathrm{cm}}^{-2}$ irradiance, is restricted to lightly doped wafers with $N_{D}≲10^{15}\;{\mathrm{cm}}^{-3}$, as established by the detection-threshold and power analyses of Section 2.4 and Section 4.4.

These limitations define the intended scope of the methodology rather than represent methodological inconsistencies. Taken together with the internal Van der Pauw consistency check, the magnetic-field stability, the Hall-factor sensitivity analysis, and the literature-based doping-range comparison reported below, they delimit a coherent and clearly bounded operating regime within which the proposed system can be applied as an accessible, low-cost tool for comparative photoconductive screening of doped *n*-type silicon, rather than as a substitute for calibrated photoconductance or carrier-resolved photo-Hall techniques.

## 5. Conclusions

A low-complexity steady-state Hall-based estimator for comparative analysis of the majority-carrier photoconductive response was implemented and experimentally assessed as a screening tool on *n*-type crystalline silicon wafers. The approach combines standard Van der Pauw and Hall measurements under dark and continuous illumination conditions, enabling extraction of an apparent Hall-derived majority-carrier metric using permanent magnets, a white LED source, and a source–measure unit.

Dark/light cycling over three independent mountings and five cycles per mounting produced mean values of $\Delta \sigma_{n,H}=29.61\pm 3.406\;\mathrm{mS}\;{\mathrm{cm}}^{-1}$ for wafer 1 and $1.167\pm 0.140\;\mathrm{mS}\;{\mathrm{cm}}^{-1}$ for wafer 2, where the ± values corresponded to the sample standard deviation over 15 cycles. These statistics quantify the short-term cycling and remounting repeatability of the two-state protocol, while long-term reproducibility remains outside the scope of the present study.

An internal consistency check based on the longitudinal Van der Pauw conductivity change showed close agreement with the Hall-derived estimator for wafer 1, whereas wafer 2 remained below the instrumental detection threshold due to its very small relative conductivity change. The lightly doped wafer exhibited a clearly resolved Hall-derived photoconductive response under illumination, while the heavily doped wafer showed a substantially reduced nominal response below the instrumental detection threshold under low-injection conditions. This contrast is consistent with the expected qualitative influence of dopant concentration; however, no quantitative wafer-to-wafer response ratio is claimed because wafer 2 lies below the instrumental detection threshold according to the SNR criterion.

Comparison with the Macdonald–Cuevas model provided a qualitative physical-consistency check, indicating that the proposed estimator follows the expected dependence on irradiance and doping concentration. Systematic deviations in absolute magnitude were observed and are attributed to the simplified treatment of minority carriers, transport effects, trapping phenomena, and the depth-dependent optical generation profile inherent to broadband illumination. This comparison should not be interpreted as a calibrated validation of the method.

The main advantage of the proposed approach is its simplicity and accessibility, making it suitable for comparative photoconductive screening and trend analysis in low-resource laboratory environments. Nevertheless, the method does not replace calibrated photoconductance techniques or carrier-resolved approaches such as SSPC, QSSPC, and photo-Hall, which provide more complete physical modeling and carrier separation. The contribution is a specific low-cost system implementation and a defined operating scope, not a new measurement principle; the present dataset cannot establish absolute accuracy relative to calibrated photoconductance or carrier-resolved photo-Hall methods.

In practical terms, the method yields a resolved response only when the injection ratio satisfies $\Delta n_{H}/N_{D}≳0.013$ (equivalently $N_{D}≲10^{15}\;{\mathrm{cm}}^{-3}$ at the present irradiance for carrier lifetimes comparable to wafer 1) under temperature control of ±0.5K; heavily doped wafers ($N_{D}≳10^{16}\;{\mathrm{cm}}^{-3}$) fall below the instrumental detection threshold at sub-$\mathrm{mW}\;{\mathrm{cm}}^{-2}$ irradiance, as quantified in Section 2.4 and the power analysis of Section 4.4.

The literature-based comparison with QSSPC, LED-QSSPC, Optical Hall, and carrier-resolved photo-Hall studies supports the qualitative consistency of the observed doping-dependent trend and clarifies the limits of direct comparison in terms of lifetime, injection regime, and photoconductive magnitude. Because independent QSSPC, SSPC, μ-PCD, or carrier-resolved photo-Hall measurements were not available for the same wafers, the reported results should be regarded as internally consistent, trend-level estimates rather than calibrated photoconductivity measurements.

Future work will focus on extending the proposed steady-state Hall-based approach to broader temperature and irradiance ranges in order to evaluate its robustness under different injection regimes. Additionally, incorporating a more detailed optical generation model and recombination analysis may improve quantitative comparison with established photoconductance techniques. Further benchmarking against calibrated QSSPC, SSPC, μ-PCD, or carrier-resolved photo-Hall measurements on the same samples would also allow a rigorous assessment of accuracy and applicability.

## Figures and Tables

**Table 1:** Position of the present Hall-derived screening system within the landscape of established photoconductance and photo-Hall techniques. The first row distinguishes the methodological status of each entry; subsequent rows summarize excitation, what is resolved, instrumentation, relative cost/complexity, and output.

	This Work	QSSPC/μ-PCD	Carrier-Resolved Photo-Hall	CLIMAT
Methodological status	System implementation; comparative screening role	Established calibrated technique	Established carrier-resolved technique	Established carrier-resolved technique
Excitation	Continuous white LED	Flash/modulated	a.c. field, modulated light	Constant light, swept field
Resolves	Majority-carrier indicator only	Effective lifetime	*n*, *p*, $\mu_{n}$, $\mu_{p}$, τ, $L_{D}$	*n*, *p*, mobilities, τ
Instrumentation	SMU + permanent magnets	rf bridge/microwave	Rotating dipole-line + lock-in	Electromagnet + lock-in
Relative cost/complexity	Low	Moderate	High	High
Output	Comparative trend/screening	Calibrated τ	Full carrier transport	Full carrier transport

**Table 2:** Repeatability and stability indicators obtained from magnetic-field and current sweeps. SD: sample standard deviation.

Quantity	Sample	*N*	Mean	SD	CV (%)
$\mu_{H}$ (${\mathrm{cm}}^{2}\;V^{-1}\;s^{-1}$), field sweep	Wafer 1	6	1335.48	1.640	0.1228
$\mu_{H}$ (${\mathrm{cm}}^{2}\;V^{-1}\;s^{-1}$), field sweep	Wafer 2	6	797.44	0.979	0.1228
$\Delta n_{H}$ (${\mathrm{cm}}^{-3}$), field sweep	Wafer 1	6	$1.384\times 10^{14}$	$3.134\times 10^{11}$	0.2264
$\Delta n_{H}$ (${\mathrm{cm}}^{-3}$), field sweep	Wafer 2	6	$9.137\times 10^{12}$	$2.069\times 10^{10}$	0.2264
$\Delta \sigma_{n,H}$ ($\mathrm{mS}\;{\mathrm{cm}}^{-1}$), field sweep	Wafer 1	6	29.617	0.0525	0.1772
$\Delta \sigma_{n,H}$ ($\mathrm{mS}\;{\mathrm{cm}}^{-1}$), field sweep	Wafer 2	6	1.167	0.00214	0.1834
$\rho_{\mathrm{dark}}$ (Ωcm), current sweep	Wafer 1	10	16.1548	0.0283	0.1749
$\rho_{\mathrm{light}}$ (Ωcm), current sweep	Wafer 1	10	10.9300	0.0283	0.2586
$\rho_{\mathrm{dark}}$ (Ωcm), current sweep	Wafer 2	10	0.081183	$1.345\times 10^{-4}$	0.1657
$\rho_{\mathrm{light}}$ (Ωcm), current sweep	Wafer 2	10	0.081175	$1.345\times 10^{-4}$	0.1657
$\mu_{H,\mathrm{light}}$ (${\mathrm{cm}}^{2}\;V^{-1}\;s^{-1}$), field sweep	Wafer 1	6	1323.62	0.962	0.0727
$\mu_{H,\mathrm{light}}$ (${\mathrm{cm}}^{2}\;V^{-1}\;s^{-1}$), field sweep	Wafer 2	6	835.38	0.0002	<0.0001

Note: The illuminated Hall mobility $\mu_{H,\mathrm{light}}$ was computed from $1/(q\;n_{H,\mathrm{light}})$ and $\rho_{\mathrm{light}}$ using the same field-sweep protocol as $\mu_{H,\mathrm{dark}}$. The difference between $\mu_{H,\mathrm{light}}$ and $\mu_{H,\mathrm{dark}}$ was 0.89% for wafer 1 and 4.76% for wafer 2, both within the cycling reproducibility of Table 3 (CV ≈11–16%), confirming that no illumination-induced mobility change was resolved within the present experimental dispersion.

**Table 3:** Dark/light cycling and sample-remounting repeatability of the Hall-derived photoconductive estimator at $1.81\;\mathrm{mW}\;{\mathrm{cm}}^{-2}$.

Sample	Mount.	Cycles	Mean $\Delta n_{H}$	SD $\Delta n_{H}$	${\mathrm{CV}}_{\Delta n}$	Mean $\Delta \sigma_{n,H}$	SD $\Delta \sigma_{n,H}$	${\mathrm{CV}}_{\Delta \sigma }$
			(${\mathrm{cm}}^{-3}$)	(${\mathrm{cm}}^{-3}$)	(%)	($\mathrm{mS}\;{\mathrm{cm}}^{-1}$)	($\mathrm{mS}\;{\mathrm{cm}}^{-1}$)	(%)
Wafer 1	1	5	$1.487\times 10^{14}$	$1.627\times 10^{13}$	10.94	31.151	2.426	7.79
Wafer 1	2	5	$1.482\times 10^{14}$	$1.886\times 10^{13}$	12.73	31.076	2.813	9.05
Wafer 1	3	5	$1.183\times 10^{14}$	$2.113\times 10^{13}$	17.86	26.615	3.151	11.84
Wafer 1	Total	15	$1.384\times 10^{14}$	$2.284\times 10^{13}$	16.50	29.614	3.406	11.50
Wafer 2	1	5	$9.221\times 10^{12}$	$1.693\times 10^{12}$	18.36	1.177	0.192	16.33
Wafer 2	2	5	$8.717\times 10^{12}$	$5.867\times 10^{11}$	6.73	1.120	0.067	5.95
Wafer 2	3	5	$9.472\times 10^{12}$	$1.322\times 10^{12}$	13.95	1.205	0.150	12.45
Wafer 2	Total	15	$9.137\times 10^{12}$	$1.233\times 10^{12}$	13.50	1.167	0.140	12.00

**Table 4:** Representative instrumental and systematic uncertainty contributions considered in the Hall-based photoconductive estimation.

Contribution	Estimated Uncertainty	Treatment
Magnetic-field calibration, *B*	∼0.2%	Instrumental RSS
Current, *I*	∼0.05%	Instrumental RSS
Thickness, *t*	1μm	Instrumental RSS
Hall-voltage repeatability, $V_{H}$	included in $R_{H}$ RSS	Instrumental RSS
Hall coefficient, $R_{H}$	0.28–0.30%	Instrumental RSS only
Carrier density, $n_{H}$	∼0.6%	Expanded instrumental uncertainty, k=2
Hall factor, $r_{H}$	0.9–1.3 sensitivity range	Systematic; see Table 5
LED temporal instability (per 3–5s cycle)	1.87%	Illumination-condition uncertainty
Spatial irradiance variation	7.46%	Illumination-condition uncertainty
Thermal drift	not independently quantified	Systematic uncertainty
Sample remounting and dark/light cycling	three mountings, five cycles each; see Table 3	Short-term reproducibility; residual long-term systematic uncertainty
Contact/alignment effects	not independently quantified	Systematic uncertainty
Magnetic-field spatial non-uniformity	not separately quantified	Partly assessed by field mapping
Parasitic photovoltage residuals	mitigated by field reversal	Systematic uncertainty

**Table 5:** Sensitivity of Hall-derived quantities to the assumed Hall factor. Values are obtained by scaling the nominal $r_{H}=1$ results. For wafer 1, the rows $r_{H}=1.10$ and 1.15 correspond to the physically expected range for $N_{D}\approx 2.9\times 10^{14}\;{\mathrm{cm}}^{-3}$ from del Alamo and Swanson [33].

Sample	$r_{H}$	$n_{H,\mathrm{dark}}$ (${\mathrm{cm}}^{-3}$)	$n_{H,\mathrm{light}}$ (${\mathrm{cm}}^{-3}$)	$\Delta n_{H}$ (${\mathrm{cm}}^{-3}$)	$\Delta \sigma_{n,H}$ ($\mathrm{mS}\;{\mathrm{cm}}^{-1}$)
Wafer 1	0.90	$2.64\times 10^{14}$	$3.88\times 10^{14}$	$1.24\times 10^{14}$	26.65
Wafer 1	1.00	$2.93\times 10^{14}$	$4.31\times 10^{14}$	$1.38\times 10^{14}$	29.61
Wafer 1	1.05	$3.08\times 10^{14}$	$4.52\times 10^{14}$	$1.45\times 10^{14}$	31.09
Wafer 1	1.10	$3.22\times 10^{14}$	$4.74\times 10^{14}$	$1.52\times 10^{14}$	32.57
Wafer 1	1.15	$3.37\times 10^{14}$	$4.96\times 10^{14}$	$1.59\times 10^{14}$	34.05
Wafer 1	1.20	$3.52\times 10^{14}$	$5.17\times 10^{14}$	$1.66\times 10^{14}$	35.53
Wafer 1	1.30	$3.81\times 10^{14}$	$5.60\times 10^{14}$	$1.79\times 10^{14}$	38.49
Wafer 2	0.90	$8.283\times 10^{16}$	$8.284\times 10^{16}$	$8.22\times 10^{12}$	1.050
Wafer 2	1.00	$9.203\times 10^{16}$	$9.204\times 10^{16}$	$9.13\times 10^{12}$	1.167
Wafer 2	1.20	$1.104\times 10^{17}$	$1.105\times 10^{17}$	$1.10\times 10^{13}$	1.400
Wafer 2	1.30	$1.196\times 10^{17}$	$1.197\times 10^{17}$	$1.19\times 10^{13}$	1.517

**Table 6:** Summary of Hall-derived parameters and photoconductive estimates for the two *n*-type silicon wafers under LED illumination at $1.81\;\mathrm{mW}\;{\mathrm{cm}}^{-2}$.

Parameter	Wafer 1	Wafer 2
Thickness, *t* (μm)	525±1	500±1
Dark resistivity, $\rho_{\mathrm{dark}}$ (Ωcm)	16.1548	0.0811829
Estimated donor concentration, $N_{D}$ (${\mathrm{cm}}^{-3}$)	$2.90\times 10^{14}$	$9.66\times 10^{16}$
$n_{H,\mathrm{dark}}$ (${\mathrm{cm}}^{-3}$)	$2.93\times 10^{14}$	$9.203\times 10^{16}$
$n_{H,\mathrm{light}}$ (${\mathrm{cm}}^{-3}$)	$4.31\times 10^{14}$	$9.204\times 10^{16}$
Apparent Hall-derived carrier-density change, $\Delta n_{H}$ (${\mathrm{cm}}^{-3}$)	$1.38\times 10^{14}$	$9.13\times 10^{12}$
Injection ratio, $\Delta n_{H}/N_{D}$	0.48±0.03	$9.5\times 10^{-5}$
Dark Hall mobility, $\mu_{H,\mathrm{dark}}$ (${\mathrm{cm}}^{2}\;V^{-1}\;s^{-1}$)	1335.48	797.44
Nominal Hall-derived estimator, $\Delta \sigma_{n,H}$ ($\mathrm{mS}\;{\mathrm{cm}}^{-1}$)	29.61	1.167
Bipolar sensitivity estimate, $\Delta \sigma_{\mathrm{bip}}$ ($\mathrm{mS}\;{\mathrm{cm}}^{-1}$)	40.0	1.48
Injection regime	Moderate (transitional) injection	Low injection; below-threshold nominal signal
${\mathrm{SNR}}_{\Delta n}$	∼88	∼$2.3\times 10^{-2}$
Detection classification	Resolved	Below threshold; trend-level nominal estimate

**Table 7:** Internal consistency check between the longitudinal Van der Pauw photoconductive response and the Hall-derived majority-carrier estimator.

Parameter	Wafer 1	Wafer 2
$\rho_{\mathrm{dark}}$ (Ωcm)	16.1548	0.0811829
$\rho_{\mathrm{light}}$ (Ωcm)	10.9300	0.0811752
$\sigma_{\mathrm{dark}}^{\mathrm{VdP}}$ ($S\;{\mathrm{cm}}^{-1}$)	0.06190	12.3179
$\sigma_{\mathrm{light}}^{\mathrm{VdP}}$ ($S\;{\mathrm{cm}}^{-1}$)	0.09149	12.3191
$\Delta \sigma_{\mathrm{VdP}}$ ($\mathrm{mS}\;{\mathrm{cm}}^{-1}$)	29.59	1.165
$\Delta \sigma_{n,H}$ ($\mathrm{mS}\;{\mathrm{cm}}^{-1}$)	29.61	1.167
$\Delta \sigma_{n,H}/\Delta \sigma_{\mathrm{VdP}}$	1.001	1.002

**Table 8:** Normalized metrics from the present Hall-based measurements used for comparison with the literature.

Parameter	Wafer 1	Wafer 2
Estimated donor concentration, $N_{D}$ (${\mathrm{cm}}^{-3}$)	$2.90\times 10^{14}$	$9.66\times 10^{16}$
Apparent Hall-derived carrier-density change, $\Delta n_{H}$ (${\mathrm{cm}}^{-3}$)	$1.38\times 10^{14}$	$9.13\times 10^{12}$
Injection ratio, $\Delta n_{H}/N_{D}$	$4.8\times 10^{-1}$	$9.5\times 10^{-5}$
Dark longitudinal conductivity, $\sigma_{\mathrm{dark}}^{\mathrm{VdP}}$ ($S\;{\mathrm{cm}}^{-1}$)	0.06190	12.3179
Hall-derived estimator, $\Delta \sigma_{n,H}$ ($\mathrm{mS}\;{\mathrm{cm}}^{-1}$)	29.61	1.167
Van der Pauw conductivity change, $\Delta \sigma_{\mathrm{VdP}}$ ($\mathrm{mS}\;{\mathrm{cm}}^{-1}$)	29.59	1.165
Relative conductivity change, $\Delta \sigma_{n,H}/\sigma_{\mathrm{dark}}^{\mathrm{VdP}}$	$4.78\times 10^{-1}$	$9.47\times 10^{-5}$
Interpretation	Resolved moderate-injection response	Below-threshold nominal low-injection estimate

**Table 9:** Quantitative comparison with reported photoconductance, lifetime, and photo-Hall studies on crystalline silicon.

Reference	Material/Doping	Technique	Injection/Light Regime	Reported Lifetime or Photoconductive Metric	Comparability with This Work
Yoon et al. [45]	Phosphorus-doped *n*-type Cz-Si; $N_{D}\approx 9.2\times 10^{14}\;{\mathrm{cm}}^{-3}$; ρ≈5Ωcm; t≈200μm	QSSPC and RCPCD	Injection-dependent lifetime measurements; low-to-moderate injection range	Initial high lifetime of approximately 2.9ms before Ni contamination; lifetime degradation analyzed through surface recombination velocity	Comparable mainly to wafer 1 in dopant concentration and injection regime. Not directly comparable in absolute magnitude because the reported quantity is minority-carrier lifetime, not $\Delta \sigma_{n,H}$, and the samples include contamination/passivation effects.
Richter et al. [37]	High-purity *n*- and *p*-type crystalline Si; $N_{\mathrm{dop}}=4.6\times 10^{13}$–$2.5\times 10^{17}\;{\mathrm{cm}}^{-3}$; wafer thickness 180–300μm	QSSPL, SSPL, PCD, and QSSPC	Injection-dependent recombination over broad dopant and excess-carrier ranges	Maximum $\tau_{\mathrm{eff}}$ decreases strongly with dopant concentration; examples include 27ms for *n*-FZ Si at $4.4\times 10^{14}\;{\mathrm{cm}}^{-3}$, 1.2ms for *n*-Cz Si at $1.3\times 10^{16}\;{\mathrm{cm}}^{-3}$, and 0.037ms for heavily doped Si at $2.5\times 10^{17}\;{\mathrm{cm}}^{-3}$	Covers the doping range of both wafers and supports the expected decrease in carrier lifetime and photoconductive response with increasing dopant concentration. Absolute values are not directly comparable because the study used highly passivated lifetime samples and calibrated lifetime techniques.
Roller et al. [46]	Photovoltaic-grade Si wafers with different resistivities, thicknesses, and passivation conditions	LED-based QSSPC	Narrow-band LED excitation over different wavelengths and intensities; below 1 sun conditions	Effective lifetime measured as a function of wavelength and intensity; reported sensitivity to generation depth, bulk lifetime, and surface recombination velocity	Highly relevant to the present white-LED excitation because it shows that spectral content and absorption depth affect the measured carrier response. Not directly comparable in magnitude because it reports $\tau_{\mathrm{eff}}$, not Hall-derived $\Delta n_{H}$ or $\Delta \sigma_{n,H}$.
Krisztián et al. [13]	Solar-cell-grade *n*-type c-Si wafers	Combined transient PC, SS-PC, and SP-PCD (single system)	Injection-dependent; integrated multi-mode operation	Simultaneous carrier concentration, mobility, and effective lifetime, with mutual agreement across the three modes	The most direct recent analogue in measured quantities (concentration, mobility, lifetime). Not directly comparable in absolute magnitude because it reports calibrated steady-state and small-perturbation lifetimes/mobilities, whereas this work reports a single steady-state Hall-derived majority-carrier indicator.
Uprety et al. [47]	Industrial Al-BSF Si solar cell; *n*-type emitter with $N_{D}=\left(3.6\pm 0.1\right)\times 10^{18}\;{\mathrm{cm}}^{-3}$ and *p*-type bulk with $N_{A}=\left(7.6\pm 0.1\right)\times 10^{15}\;{\mathrm{cm}}^{-3}$	Optical Hall effect	Dark and 1 sun AM1.5 illumination	Under 1 sun, photogenerated minority-carrier concentrations of $\Delta p=\left(7.8\pm 0.2\right)\times 10^{16}\;{\mathrm{cm}}^{-3}$ in the *n*-type emitter and $\Delta n=\left(2.2\pm 0.2\right)\times 10^{14}\;{\mathrm{cm}}^{-3}$ in the *p*-type bulk were reported	Useful as a carrier-resolved Hall-type benchmark. Not directly comparable because it uses a complete solar cell, optical Hall analysis, much higher illumination, and a heavily doped emitter rather than bare *n*-type wafers.
Gunawan et al. [11]	Single-crystal Si and photovoltaic absorber materials	Carrier-resolved photo-Hall	Light-intensity-dependent photo-Hall measurements	Simultaneous extraction of majority/minority carrier density, mobility, lifetime, diffusion length, and recombination coefficient	Methodologically comparable because it extends Hall measurements under illumination. Not quantitatively equivalent because the present work does not resolve electron and hole contributions separately and uses a simpler steady-state Hall estimator.
Musiienko et al. [18]	Si and other semiconductors; reported Si demonstration includes carrier-resolved transport parameters	CLIMAT	Constant-light magnetotransport under controlled illumination	Resolves electron and hole mobility, density, lifetime, diffusion coefficient, diffusion length, and quasi-Fermi-level splitting	Strong methodological contrast. It supports the limitation statement that simplified illuminated Hall measurements cannot replace carrier-resolved methods. Quantitative comparison is limited because CLIMAT separates carrier populations whereas $\Delta \sigma_{n,H}$ is a majority-carrier estimator.

## Data Availability

The raw Hall-voltage, resistivity, magnetic-field, irradiance, dark/light cycling, remounting, and processed data used in this study are available from the corresponding author upon reasonable request.

## Supplementary Materials

The following supporting information can be downloaded at: https://www.mdpi.com/article/10.3390/ma19143016/s1, Table S1. Individual dark/light cycling values used to calculate the repeatability statistics in Table 3.

## Abbreviations and Nomenclature

The following abbreviations are used in this manuscript:

SSPC	steady-state photoconductance
QSSPC	quasi-steady-state photoconductance
CRPH	carrier-resolved photo-Hall
CLIMAT	constant light-induced magnetotransport
VdP	Van der Pauw
SNR	signal-to-noise ratio
RCA	Radio Corporation of America (standard wafer cleaning procedure)

Nomenclature and units used in this work.

**Symbol**	**Description**	**Units**
Δσ	Full bipolar photoconductivity change, including electron and hole contributions	$S\;{\mathrm{cm}}^{-1}$
$\Delta \sigma_{n,H}$	Hall-derived majority-carrier photoconductive estimator	$S\;{\mathrm{cm}}^{-1}$
$\Delta \sigma_{n,H}^{\left(1\right)}$	Nominal Hall-derived photoconductive estimator calculated using $r_{H}=1$	$S\;{\mathrm{cm}}^{-1}$
$\Delta \sigma_{\mathrm{bip}}$	Bipolar sensitivity estimate obtained by including an approximate hole contribution	$S\;{\mathrm{cm}}^{-1}$
$\Delta \sigma_{\mathrm{VdP}}$	Longitudinal photoconductive response estimated from Van der Pauw resistivity	$S\;{\mathrm{cm}}^{-1}$
Δn, Δp	Excess electron and hole densities in the general photoconductivity expression	${\mathrm{cm}}^{-3}$
$\Delta n_{H}$	Illumination-induced change in Hall-derived majority-carrier density	${\mathrm{cm}}^{-3}$
$\Delta n_{H}^{\left(1\right)}$	Nominal illumination-induced Hall-derived carrier-density change calculated using $r_{H}=1$	${\mathrm{cm}}^{-3}$
$n_{H}$	Hall-derived majority-carrier density	${\mathrm{cm}}^{-3}$
$n_{H}^{\left(1\right)}$	Nominal Hall-derived majority-carrier density calculated using $r_{H}=1$	${\mathrm{cm}}^{-3}$
$n_{H,\mathrm{dark}}$, $n_{H,\mathrm{light}}$	Hall-derived carrier density under dark and illuminated conditions	${\mathrm{cm}}^{-3}$
$N_{D}$	Estimated donor concentration	${\mathrm{cm}}^{-3}$
$\mu_{n}$, $\mu_{p}$	Electron and hole mobilities	${\mathrm{cm}}^{2}\;V^{-1}\;s^{-1}$
$\mu_{H,\mathrm{dark}}$	Dark Hall electron mobility used in the estimator	${\mathrm{cm}}^{2}\;V^{-1}\;s^{-1}$
$r_{H}$	Hall factor	dimensionless
ρ	Electrical resistivity	Ωcm
$\rho_{\mathrm{dark}}$, $\rho_{\mathrm{light}}$	Dark and illuminated Van der Pauw resistivity	Ωcm
$\sigma_{\mathrm{dark}}^{\mathrm{VdP}}$, $\sigma_{\mathrm{light}}^{\mathrm{VdP}}$	Dark and illuminated longitudinal conductivity obtained from Van der Pauw resistivity	$S\;{\mathrm{cm}}^{-1}$
τ	Carrier lifetime	s
$R_{H}$	Hall coefficient	${\mathrm{cm}}^{3}\;C^{-1}$
$V_{H}$	Hall voltage	V
*t*	Wafer thickness	μm
*I*	Applied current	μA
*B*	Magnetic field	T
${\mathrm{SNR}}_{\Delta n}$	Signal-to-noise ratio of the apparent Hall-derived carrier-density change	dimensionless
$u_{c}\left(\Delta n_{H}\right)$	Combined standard uncertainty of the apparent Hall-derived carrier-density change	${\mathrm{cm}}^{-3}$
$u_{c}\left(n_{H}\right)$	Combined standard uncertainty of the Hall-derived carrier density	${\mathrm{cm}}^{-3}$
