# Supplementary material for: A Low-Complexity Hall-Based Measurement System Implementing a Dark/Illuminated Differential Estimator for Majority-Carrier Photoconductive Screening in Doped n-Type Silicon
Source: Materials (Basel). 2026 Jul 13;19(14):3016. doi: 10.3390/ma19143016 (PMC13413338; doi:10.3390/ma19143016)
Supplement: Supplementary file 1 [file materials-19-03016-s001.zip › materials-4364995-supplementary.pdf]

materials

Article

A Low-Complexity Hall-Based Measurement System Implementing a Dark/Illuminated Differential Estimator for Majority-Carrier Photoconductive Screening in Doped *n*-Type Silicon

Bernardo Reyes-Durán [1], Carlos Álvarez-Macías [1,*], Lizbeth Salgado-Conrado [2], Alma Esmeralda-Gómez [2] and Raúl Tadeo-Rosas [2]

1 Tecnológico Nacional de México/Instituto Tecnológico de La Laguna, Torreón 27000, Coahuila, Mexico
2 Facultad de Ingeniería Mecánica y Eléctrica, Universidad Autónoma de Coahuila, Carretera Torreón-Matamoros, km 7.5, Torreón 27276, Coahuila, Mexico; lizbeth_salgado@uadec.edu.mx (L.S.-C.)
* Correspondence: calvarezm@correo.itlalaguna.edu.mx

Table S1. Individual dark/light cycling values used to calculate the repeatability statistics in Table 3.

Sample	Mount.	Cycle	$n_{H,\text{dark}}$ (cm^{-3})	$n_{H,\text{light}}$ (cm^{-3})	Δn_H (cm^{-3})	$\Delta\sigma_{n,H}$ (mS cm^{-1})
Wafer 1	1	1	2.93007×10^{14}	4.42597×10^{14}	1.496×10^{14}	31.280
Wafer 1	1	2	2.93007×10^{14}	4.28007×10^{14}	1.350×10^{14}	29.105
Wafer 1	1	3	2.93007×10^{14}	4.46067×10^{14}	1.531×10^{14}	31.797
Wafer 1	1	4	2.93007×10^{14}	4.66177×10^{14}	1.732×10^{14}	34.796
Wafer 1	1	5	2.93007×10^{14}	4.25807×10^{14}	1.328×10^{14}	28.776
Wafer 2	1	1	9.20318×10^{16}	9.20427×10^{16}	1.093×10^{13}	1.371
Wafer 2	1	2	9.20318×10^{16}	9.20418×10^{16}	9.981×10^{12}	1.263
Wafer 2	1	3	9.20318×10^{16}	9.20383×10^{16}	6.492×10^{12}	0.867
Wafer 2	1	4	9.20318×10^{16}	9.20407×10^{16}	8.864×10^{12}	1.136
Wafer 2	1	5	9.20318×10^{16}	9.20416×10^{16}	9.836×10^{12}	1.247
Wafer 1	2	1	2.93007×10^{14}	4.25807×10^{14}	1.328×10^{14}	28.776
Wafer 1	2	2	2.93007×10^{14}	4.67467×10^{14}	1.745×10^{14}	34.988
Wafer 1	2	3	2.93007×10^{14}	4.48817×10^{14}	1.558×10^{14}	32.208
Wafer 1	2	4	2.93007×10^{14}	4.20407×10^{14}	1.274×10^{14}	27.970
Wafer 1	2	5	2.93007×10^{14}	4.43657×10^{14}	1.506×10^{14}	31.437
Wafer 2	2	1	9.20318×10^{16}	9.20396×10^{16}	7.765×10^{12}	1.012
Wafer 2	2	2	9.20318×10^{16}	9.20411×10^{16}	9.287×10^{12}	1.184
Wafer 2	2	3	9.20318×10^{16}	9.20408×10^{16}	9.044×10^{12}	1.157
Wafer 2	2	4	9.20318×10^{16}	9.20407×10^{16}	8.883×10^{12}	1.138
Wafer 2	2	5	9.20318×10^{16}	9.20404×10^{16}	8.607×10^{12}	1.107
Wafer 1	3	1	2.93007×10^{14}	4.20537×10^{14}	1.275×10^{14}	27.991
Wafer 1	3	2	2.93007×10^{14}	4.20487×10^{14}	1.275×10^{14}	27.983
Wafer 1	3	3	2.93007×10^{14}	4.36747×10^{14}	1.437×10^{14}	30.407
Wafer 1	3	4	2.93007×10^{14}	3.87234×10^{14}	9.423×10^{13}	23.024
Wafer 1	3	5	2.93007×10^{14}	3.91561×10^{14}	9.855×10^{13}	23.670
Wafer 2	3	1	9.20318×10^{16}	9.20413×10^{16}	9.545×10^{12}	1.214
Wafer 2	3	2	9.20318×10^{16}	9.20425×10^{16}	1.073×10^{13}	1.349
Wafer 2	3	3	9.20318×10^{16}	9.20392×10^{16}	7.387×10^{12}	0.969
Wafer 2	3	4	9.20318×10^{16}	9.20423×10^{16}	1.046×10^{13}	1.318
Wafer 2	3	5	9.20318×10^{16}	9.20410×10^{16}	9.230×10^{12}	1.178

The listed $n_{H,\text{light}}$ values were calculated as $n_{H,\text{dark}} + \Delta n_H$ using the nominal dark Hall-derived baseline for each wafer. The $\Delta\sigma_{n,H}$ values are reported in mS cm^{-1}.

Check for updates

Academic Editor: Spiros Gardelis

Received: 23 May 2026
Revised: 2 July 2026
Accepted: 5 July 2026
Published: 13 July 2026

Copyright: © 2026 by the authors. Licensee MDPI, Basel, Switzerland. This article is an open access article distributed under the terms and conditions of the Creative Commons Attribution (CC BY) license.
